# Safely managed drinking water service use and child diarrhoea based on evidence from 24 countries

**DOI:** 10.1038/s44221-026-00647-4

**Published:** 2026-06-02

**Authors:** Esther E. Greenwood, Mathilda Freymond, Andreas Scheidegger, Thomas Lauber, Kamshat Tussupova, Amy J. Pickering, Timothy R. Julian

**Affiliations:** 1 Department of Environmental Systems Science, ETH Zurich, Swiss Federal Institute of Technology, Zurich, Switzerland; 2 Eawag, Swiss Federal Institute of Aquatic Science and Technology, Dübendorf, Switzerland; 3 Department of Health Sciences and Technology, ETH Zurich, Swiss Federal Institute of Technology, Zurich, Switzerland; 4 Department of Water Resources Engineering, Lund University, Lund, Sweden; 5 Kazakh National University of Water Management and Irrigation, Taraz, Kazakhstan; 6 Department of Civil and Environmental Engineering, University of California, Berkeley, Berkeley, CA USA; 7 Blum Center for Developing Economies, University of California, Berkeley, Berkeley, CA USA; 8 Biohub, San Francisco, CA USA; 9 Swiss Tropical and Public Health Institute, Allschwil, Switzerland; 10 University of Basel, Basel, Switzerland

**Keywords:** Environmental impact, Diarrhoea

## Abstract

Universal access to safe drinking water is called for under Sustainable Development Target 6.1 (SDG 6.1). Yet quantitative evidence on disease reductions associated with achieving SDG 6.1 and the service dimensions that most effectively reduce diarrhoeal diseases remains limited and inconsistent. Here, using multiple indicator cluster survey data from 24 low- and middle-income countries, we show that household use of safely managed drinking water services (SMDWS) reduces diarrhoea risks in children under 5 years old compared with use of services not fulfilling all of the SMDWS criteria. Drinking water availability when needed and absence of faecal contamination at the point of use showed consistent protective associations with child diarrhoea. We observed less evidence of the protective effect of an improved drinking water source, access on premises and absence of faecal contamination at the point of collection. Our findings reaffirm the importance of SMDWS in protecting against child diarrhoea, while highlighting uncertainties in estimates of the magnitude of the disease burden, which could be prevented by achieving SDG 6.1.

## Abstract (web summary)

Safe drinking water is vital for reducing childhood diarrhoea, but evidence on which service elements matter most has been unclear. Data from 24 low- and middle-income countries show that safely managed drinking water reduces child diarrhoea risk, with water availability and absence of faecal contamination at point of use providing the strongest protection.

## Main

Diarrhoeal disease remains a leading cause of mortality in children under 5 years old globally, reportedly causing over 440,000 deaths in 2021^1^. Despite a substantial decline in diarrhoea associated mortality since 1990, declines in rates of diarrhoeal incidence have been less pronounced^2^. Diarrhoeal incidence has been associated with unsafe drinking water, sanitation and hygiene, which commonly affects people in low- and middle-income countries (LMICs), consistent with observed inequities in the global distribution of diarrhoeal disease-associated morbidity and mortality^3,4^. Diarrhoeal disease can be caused by a range of pathogens, some of which have been shown to be influenced by weather patterns, including heavy rainfall, extended dry periods and high ambient temperatures^5,6^. Climate change, expected to further exacerbate extreme weather events, threatens to also increase diarrhoeal disease-related morbidity and mortality^7,8^.

Provision of safe drinking water is essential for safeguarding human health and, as such, was formally recognized as a human right by the United Nations (UN) in 2010^9^. The human right to water encompasses aspects of availability, physical accessibility, acceptability, affordability and quality. Universal access to safe and affordable drinking water is defined as a priority under Sustainable Development Goal target 6.1 (SDG 6.1)^10^. To facilitate monitoring of drinking water services across countries globally, the Joint Monitoring Programme for Water Supply and Sanitation (JMP) developed a service ladder focused on quantifying the quality, accessibility and availability of sufficient amounts of drinking water when needed^10,11^. Within the JMP framework, use of safely managed drinking water services (SMDWS) represents the highest level of drinking water service provision and is the global indicator SDG 6.1.1 used to monitor progress towards SDG 6.1. To be regarded as using SMDWS, households must use drinking water (1) from an improved source, (2) accessible on premises, (3) available when needed and (4) free from faecal and priority chemical contamination at the point of collection (POC)^11^. Global estimates of the number of people lacking SMDWS ranged between 2.2 billion and over 4.4 billion people as of 2020, with approximately 2 billion estimated to use drinking water contaminated with faeces^10,12,13^. The World Health Organization (WHO) recommends using risk categories for *Escherichia coli* contamination to monitor improvements in drinking water quality over time and help inform priorities for action in their drinking water guidelines^14^.

Despite global targets to provide safe drinking water services for all, quantitative evidence on disease reductions associated with achieving SDG 6.1 remains limited. Evidence on which drinking water service dimensions most effectively reduce diarrhoeal diseases also remains inconsistent. Previous estimates of the preventable diarrhoeal disease burden attributable to achieving SDG target 6.1 have relied on limited quantitative evidence available on exposure–response relationships from controlled trials^15^. Relative risk (RR) reductions attributable to use of SMDWS from a meta-analysis of data from controlled trials shows that higher levels of drinking water services are more effective at reducing diarrhoeal disease than lower levels^16^. However, owing to data paucity, no studies were included that evaluated the effect of interventions meeting all subcomponent criteria of SMDWS, as defined within the SDG framework^15^. Furthermore, substantial heterogeneity in effect sizes of interventions improving drinking water quality and accessibility is reported in previous controlled trials^16–18^. Understanding which dimensions of drinking water services are likely to contribute to the greatest health benefits is important so that investments in LMICs can be targeted efficiently.

The observed heterogeneity in impacts of drinking water interventions on diarrhoeal disease may be influenced by context-specific factors, including variations in weather and climate^19^. Diarrhoeal disease is environmentally transmissible and can be caused by a range of pathogens, some of which have been shown to be influenced by weather patterns^4–6^. The effectiveness of drinking water services on reducing diarrhoeal diseases may be impacted by reduced intervention efficacy as well as increased exposure to environmental contamination, which are exacerbated by weather conditions^8,20^. Childhood diarrhoea has been positively associated with low rainfall, including long-term droughts across tropical, arid and cold zones but has also been reported to peak during rainy periods^7,21,22^. Studies have suggested that differences in the direction of associations of diarrhoea and rainfall may be partially explained by the ‘concentration–dilution’ effect. The hypothesis is that pathogens accumulate in the environment during dry periods and are then flushed into drinking water sources following heavy rainfall events^8,23^. Understanding the extent to which rainfall patterns modify the effectiveness of SMDWS across climate zones is relevant for understanding climate vulnerability of drinking water service interventions.

In this study, we estimated the effect of using SMDWS on caregiver-reported childhood diarrhoea prevalence over a 2-week recall period across 24 LMICs. In a secondary analysis, we investigated the effects of four SMDWS criteria on child diarrhoea, including the use of drinking water (1) from an improved source, (2) accessible on premises, (3) available when needed and (4) free of faecal contamination at the POC (based on the absence of *E. coli* in a 100-ml drinking water sample). Finally, we assess the degree to which climate zone and heavy rainfall following a dry period modify the effect of SMDWS on diarrhoea. For all models directed acyclic graphs (DAGs) were created using the Dagitty software (v3.1) describing our assumptions on all exposure routes and confounding factors that may influence the causal relationships between SMDWS, its subcomponents and childhood diarrhoea^24^ (Supplementary Figs. 1–4). Inclusion of variables in the model was informed by prior literature highlighting their role in influencing the relationship between SMDWS and diarrhoea^8,15,20,25–39^. These included variables describing child age, child sex, household’s basic sanitation services and wealth status, living area (urban or rural) and the mother’s education level, as well as average Earth surface temperature and total precipitation (Supplementary Table 1). Data are from household and child health survey data gathered between 2016 and 2020 (Supplementary Table 2) within the framework of the United Nations Children’s Fund (UNICEF) Multiple Indicator Cluster Survey (MICS) Programme^40^. Information on total precipitation was sampled from the Climate Hazards group Infrared Precipitation with Stations (CHIRPS) dataset and average surface temperature was sampled from the European ReAnalysis (ERA5)–Land monthly averaged data^41,42^ for the area households were located in using Google Earth Engine^43^. We applied multilevel log-binomial generalized linear models with a random intercept at country level to estimate the RR of children under 5 years old having diarrhoea when using SMDWS as well as when fulfilling the subcriteria of SMDWS. Data on a single child per household were included in the analyses and children were randomly selected in cases with data on more than one child per household to avoid intrahousehold correlation.

## Summary statistics of the survey data

**Fig. 1:**
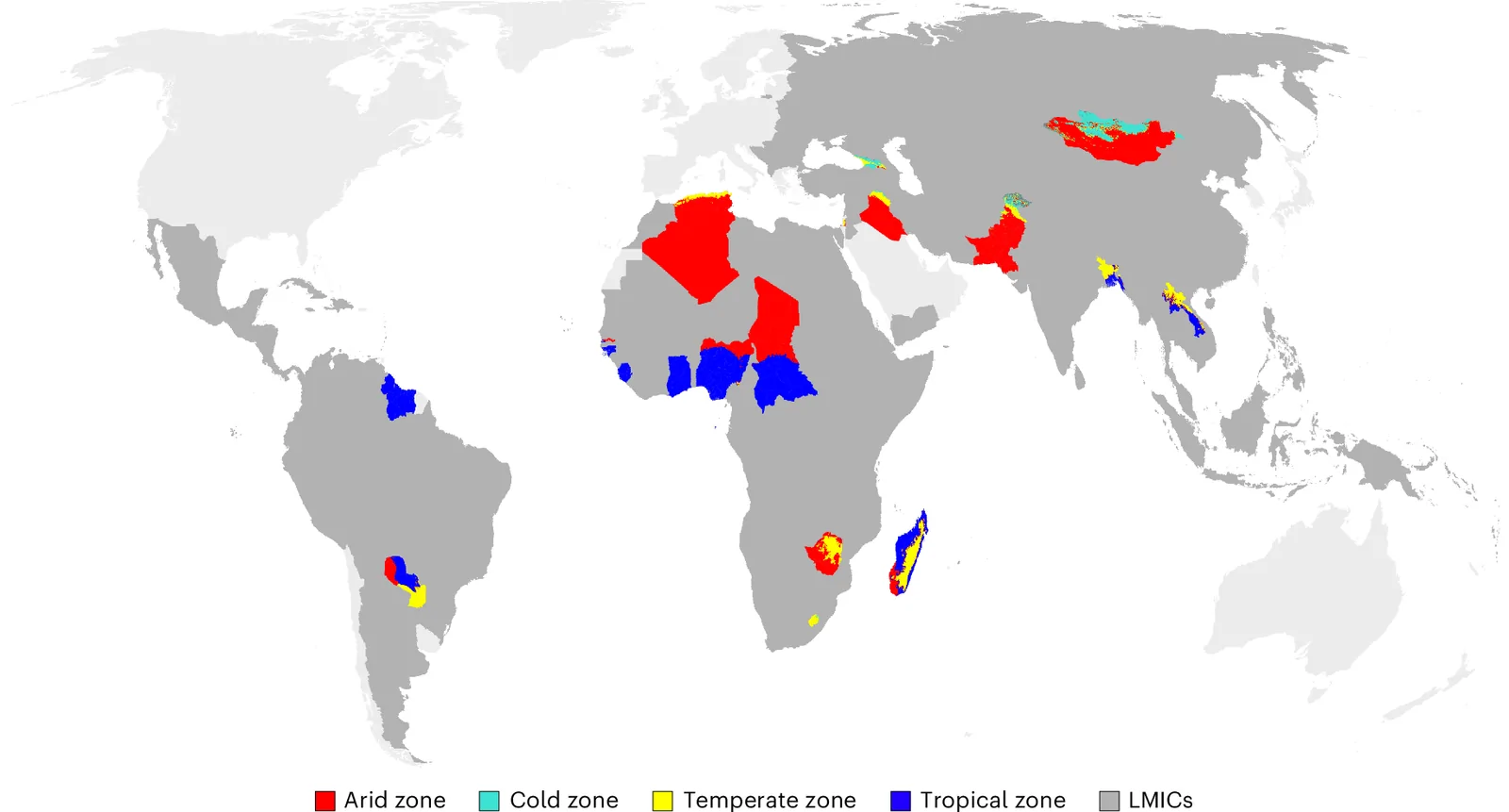
Geographic locations of surveys and associated climate zones included in our analysis. Survey locations are mapped across LMICs (shaded grey), with household data from multiple indicator cluster surveys from 24 LMICs gathered between 2016 and 2020. Arid (red), cold (turquoise), temperate (yellow) and tropical (blue) zones are classified according to Köppen–Geiger climate zone classification. Figure adapted with permission from ref. ^12^, AAAS.

From the 24 MICS surveys included in our analysis, a total of 26,796 households with children under 5 years old met the inclusion criteria, of which 13,061 were located in Africa, 12,025 in Asia and 1,710 in the Americas (Supplementary Table 1). The surveys were conducted across four Köppen–Geiger climate zones^44^: 10,617 households were located in arid zones, 213 in cold zones, 4,926 in temperate zones and 11,557 in tropical zones (Fig. 1). Diarrhoea, defined in the MICS as reported by mother or caretaker, occurred in 13.1% of the children in our study, which included a subsample of one randomly chosen child under the age of 5 years per household. Diarrhoea occurred in 13.6% of all children under 5 years (*n* = 40,407) across the 26,796 households (Supplementary Table 1). Prevalence rates among children varied in our study population, with the highest rates in the Central African Republic (23%), Chad (25%) and Gambia (26%) and the lowest rates in Algeria (4%) (Supplementary Table 1). In our study population, 27% of children lived in households using SMDWS defined by using a drinking water source which was improved, accessible on premises, with water available when needed and free of faecal contamination at the POC, measured using *E. coli* as an indicator for faecal contamination. Notably, SMDWS use dropped to 19% when absence of faecal contamination was defined based on the water quality of stored drinking water at the point of use (POU) rather than water quality directly from the source, at POC. Lack of SMDWS was primarily driven by drinking water contamination, which affected 53% of drinking water sources and 70% of stored drinking water. Drinking water accessibility was the second most relevant driver, with less than half of the observed population using drinking water sources accessible on premises. Improved drinking water sources were used by 82% of households and drinking water was available in sufficient quantities when needed in 86%.

Our main model was adjusted for child age, sex, use of basic sanitation services, type of living area (urban or rural), mother’s education level and relative wealth quintile, as well as the average 30-day Earth surface temperature and total precipitation over 30 days before survey data collection. Descriptive statistics of these population characteristics across the entire dataset, as well as stratified by climate zone, dry and wet period, and country level can be found in Supplementary Table 1.

## Risk of diarrhoea associated with lack of safe drinking water

**Fig. 2:**
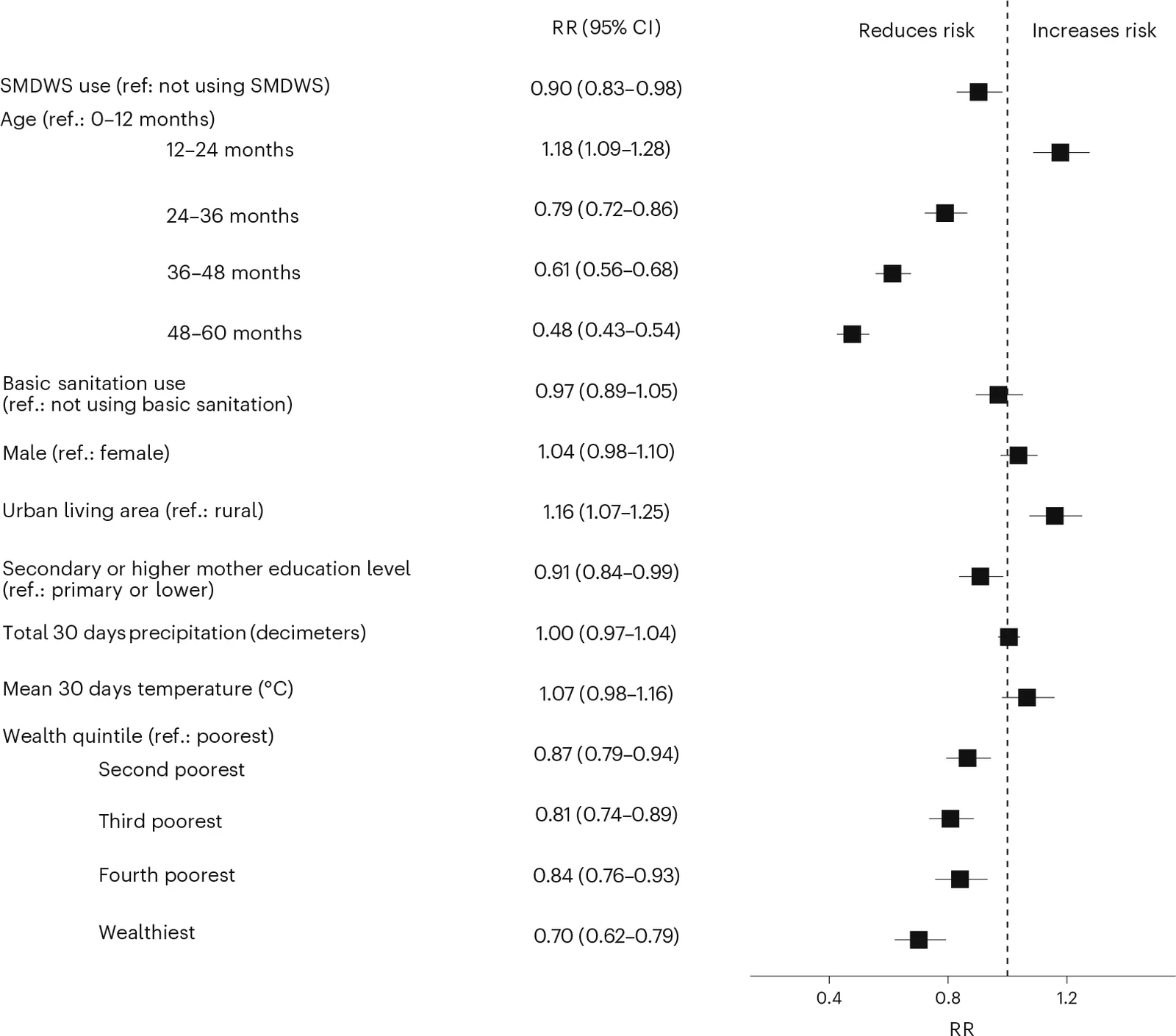
Adjusted RR for diarrhoeal disease in children living in households using SMDWS. Adjusted RR for diarrhoeal disease in children living in households using SMDWS at the POC (*n* = 7,320) compared with children living in households not fulfilling at least one SMDWS criterion (*n* = 19,476). The squares represent point estimates of RRs and the horizontal lines the 95% CIs of the primary exposure and all adjustment variables derived from a multilevel log-binomial generalized linear model. The dashed vertical line at RR of 1 marks no change in risk.

Our results highlight that household use of SMDWS contributes significantly to reducing diarrhoeal disease in children under 5 years in LMICs compared with the use of drinking water services that do not fulfil all the SMDWS criteria (RR 0.90, 95% confidence interval (CI) 0.83–0.98) (Fig. 2 and Supplementary Table 3). A greater risk reduction for childhood diarrhoea was observed when comparing children in households using SMDWS with those using drinking water services classified as less than basic (RR 0.87, 95% CI 0.78–0.97) (Supplementary Fig. 5). Redefining SMDWS to be free from faecal contamination at the POU rather than POC did not meaningfully change the RRs of child diarrhoea in relation to not fulfilling all of the SMDWS criteria (RR 0.90, 95% CI 0.81–0.99) or using services that are considered less than basic (RR 0.86, 95% CI 0.76–0.96) (Supplementary Figs. 6 and 7). Using a drinking water source that was classified as at least basic but not safely managed at the POC (RR 0.95, 95% CI 0.88–1.03) or POU (RR 0.95, 95% CI 0.88–1.02) did not significantly reduce diarrhoeal disease in children under 5 years (Supplementary Figs. 5 and 7 Supplementary Table 3).

Based on the estimated risk reductions, of the 3,503 cases of children with diarrhoea within our study population, approximately 6.8% (237, 95% CI 50–385) cases, could have been prevented through universal provision of SMDWS with drinking water free of faecal contamination at the POC. A similar proportion, 7.5% (261, 95% CI 56–416) cases, could have been prevented if drinking water was free of faecal contamination at the POU.

**Fig. 3:**
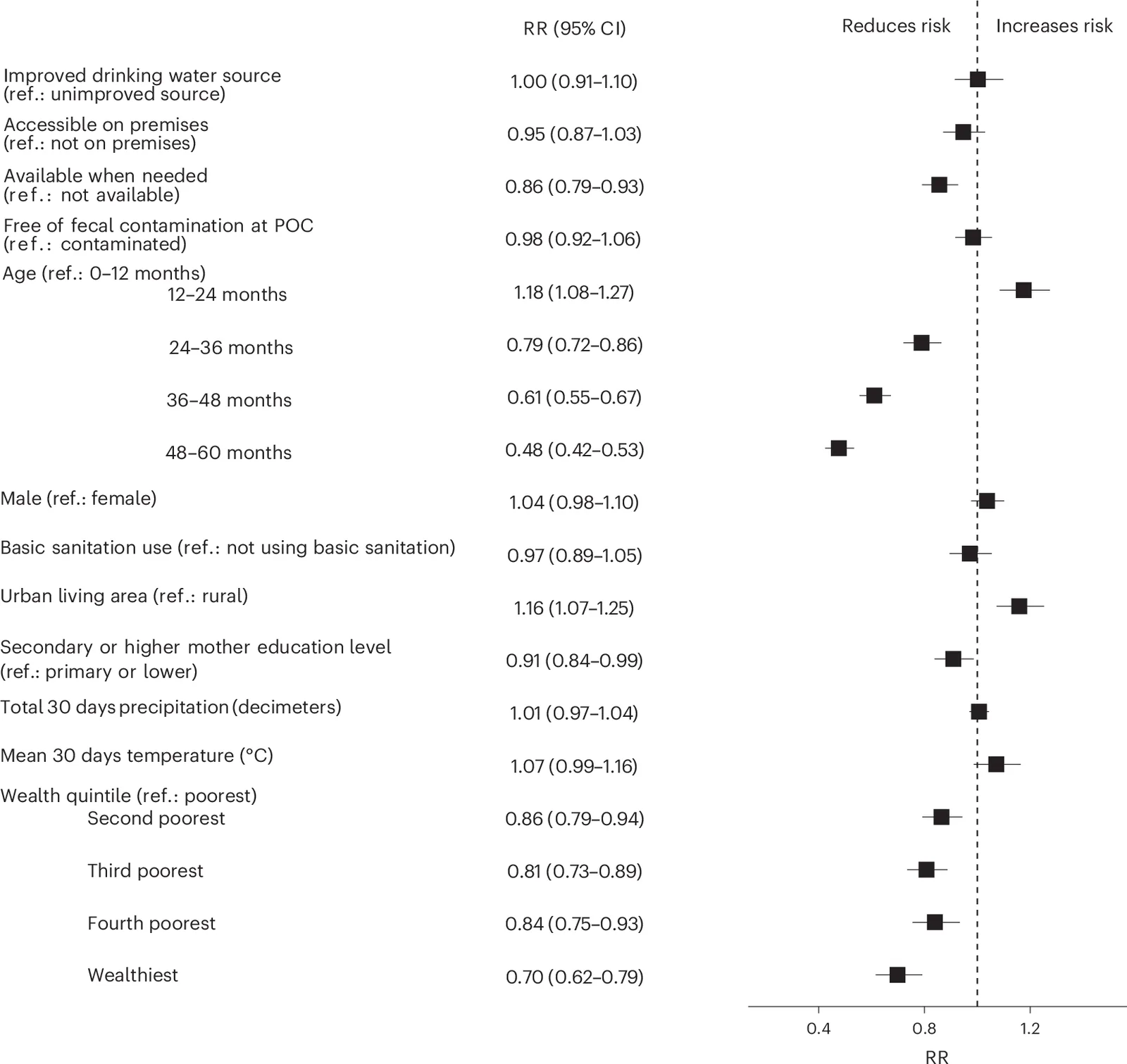
Adjusted RR for diarrhoeal disease by SMDWS subcomponents. Adjusted RR for diarrhoeal disease in children living in households using a drinking water source that was (1) improved (*n* = 22,179, unimproved *n* = 4,617), (2) accessible on premises (*n* = 14,992, not on premises *n* = 11,804), (3) available when needed (*n* = 22,901, not available *n* = 3,868) and (4) free of faecal contamination at the POC (*n* = 12,582, contaminated *n* = 14,187). The squares represent the point estimates of RRs and horizontal lines the 95% CIs of the primary exposure and all adjustment variables derived from a multilevel log-binomial generalized linear model. The dashed vertical line at RR of 1 marks no change in risk.

We conducted secondary analyses to assess the individual impact of the SMDWS subcriteria used to monitor progress towards SDG 6.1 on childhood diarrhoeal disease. The subcriteria include whether the children’s primary drinking water was (1) from an improved source, (2) accessible on premises, (3) free of faecal contamination at the POC and (4) available when needed. We observed that having drinking water available when needed had the greatest effect on the RR of a child having diarrhoea (RR 0.86, 95% CI 0.79–0.93) (Fig. 3). The household survey question included in the MICS on drinking water availability asks whether household members had experienced drinking water insufficiency within the previous month^11^. With 15% of our study population experiencing drinking water insufficiency, we estimate that approximately 2% (95% CI 1–3%) of all diarrhoeal cases were attributable to lack of drinking water availability when needed. No significant reductions in risk (RR 1.0, 95% CI 0.91–1.10) were observed for children using a drinking water source which was improved (*n* = 22,179) compared with unimproved sources (*n* = 4,617). Neither were substantial reductions observed by using piped drinking water services on premises (RR 1.01, 95% CI 0.87–1.16, *n* = 5,685) or packaged and bottled water (RR 0.9, 95% CI 0.76–1.07, *n* = 2,416). Using drinking water that was accessible on premises (RR 0.95, 95% CI 0.87–1.03) did not significantly reduce diarrhoea compared with water that needed to be collected off premises. While the absence of drinking water contamination at the POC did not impact children’s risk of diarrhoeal disease (RR 0.98, 95% CI 0.92–1.06) (Fig. 3), absence of contamination at the POU did significantly reduce the risk of childhood diarrhoeal disease (RR 0.91, 95% CI 0.83–0.98) (Supplementary Fig. 8). We estimated that using drinking water free of contamination at the POU could have prevented approximately 3% of all diarrhoea cases (95% CI 1–5%). This indicator, however, is not currently used to monitor progress towards SDG 6.1.

**Fig. 4:**
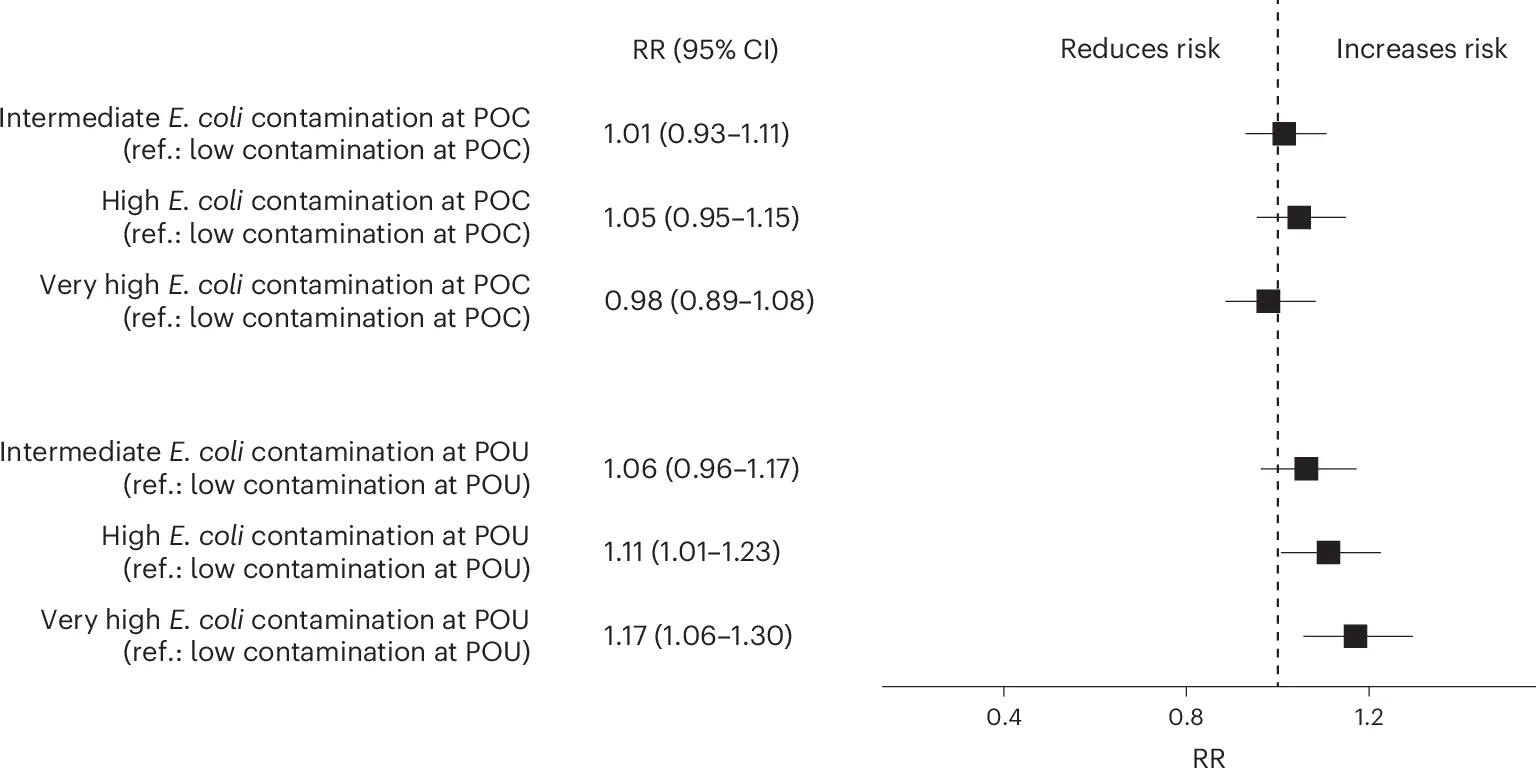
Adjusted RR for diarrhoeal disease in children based on *E. coli* contamination of drinking water. Adjusted RR for diarrhoeal disease in children living in households using drinking water with intermediate (1 < 10 CFU per 100 ml, at POC *n* = 4,549 and at POU *n* = 5,017), high (10 < 100 CFU per 100 ml, at POC *n* = 4,073 and at POU *n* = 6,058) and very high (≥100 CFU per 100 ml, at POC *n* = 5,592 and at POU *n* = 7,732) levels of *E. coli* contamination at the POC (top) and POU (bottom) compared with low levels of *E. coli* contamination (<1 CFU per 100 ml, at POC *n* = 12,582 and at POU *n* = 7989). The squares represent the point estimates of RRs and horizontal lines the 95% CIs of the primary exposure derived from multilevel log-binomial generalized linear models. The dashed vertical line at RR of 1 marks no change in risk.

The WHO recommends using risk categories for *E. coli* contamination to monitor improvements in drinking water quality over time in their drinking water guidelines to help inform priorities for action^14^. We analysed the impact of *E. coli* contamination risk categories of low (<1 colony forming units (CFU) per 100 ml), intermediate (1 < 10 CFU per 100 ml), high (10 < 100 CFU per 100 ml) and very high (≥100 CFU per 100 ml) levels of contamination, for both POC and POU^14^. When considering faecal contamination at the POU, the RR of diarrhoea increased with higher levels of contamination (Fig. 4). Compared with low levels of faecal contamination, RRs of diarrhoea significantly increased when drinking water with both high (RR 1.11, 95% CI 1.01–1.23) and very high (RR 1.17, 95% CI 1.06–1.30) levels of faecal contamination was used (Fig. 4). Notably, every increase in risk category (representing 1-log_10_ ranges in *E. coli* counts per 100 ml sample) was associated with a 4–6% increase in diarrhoeal disease risk. These findings are aligned with results from a longitudinal study that provided evidence for a causal relationship between higher levels of faecal contamination in household drinking water and increased rates of diarrhoeal disease in rural areas in Bangladesh^45^. However, we find that this relationship only holds at the POU and do not observe a significant increase in the risk of diarrhoeal disease in children from households using drinking water with intermediate (RR 1.01, 95% CI 0.93–1.11), high (RR 1.05, 95% CI 0.95–1.15) or very high (RR 0.98, 95% CI 0.89–1.08) levels of contamination at the POC. These findings suggest that improvements in drinking water quality at the POU but not the POC may be protective against children’s diarrhoeal disease, even when *E. coli* is still detected at low concentrations. Furthermore, these findings support the continued promotion of the WHO risk categories as meaningful indicators of water quality at POU and as a tool to inform priorities for action.

Based on subgroup analyses, we observed that the effect of household use of SMDWS defined at the POC on childhood diarrhoea was influenced by climate zone. The largest effect size of SMDWS was observed in tropical regions (RR 0.74, 95% CI 0.63–0.88) compared with those in temperate (RR 0.86, 95% CI 0.69–1.07) and arid regions (RR 1.0, 95% CI 0.89–1.12) (Supplementary Fig. 9). Children living in tropical climate zones were characterized with the highest diarrhoeal period prevalence rate (14.9%) as well as the lowest SMDWS use rate (12.7%) compared with those living in arid, cold and temperate climates (Supplementary Data 1). Differences in effect sizes were also observed between samples collected within 30 days of the onset of a wet period (RR 0.94, 95% CI 0.80–1.12) compared with those collected outside these conditions (RR 0.89, 95% CI 0.8–0.98) (Supplementary Fig. 10). These results are in line with results from meta-analysis of studies investigating the concentration–dilution effect on diarrhoea and suggest that rainfall following dry periods may increase the risk of diarrhoeal disease by flushing pathogens in the environment into drinking water sources^20^.

Among control variables, child age, living area and household relative wealth status had a statistically significant influence on the RR of diarrhoeal disease. Our results point towards children below 24 months and households within the poorest wealth quintile in their country being most at risk of diarrhoeal disease. These associations are well aligned with evidence from the literature on increased risks associated with child age^46^ and income level^47^ but stand in contrast to higher risks associated with living in rural areas reported in the literature^48^. However, as our model was not built to quantify causal relationships between control variables and diarrhoeal disease, caution should be taken with the interpretation of such observed effects in our study.

Sensitivity analyses indicated that our primary findings were robust to finer-scale clustering (adding a random intercept at the subnation district level) and the random selection of one child per household using a Monte Carlo simulation (Supplementary Figs. 11–14 and Supplementary Table 4). The removal of correlated exposure measures also did not affect our results: estimated RRs and CIs of childhood diarrhoea did not change compared with the main subcomponent model (Supplementary Table 5) nor did it meaningfully change for *E. coli* risk category models after removing information on water source type (Supplementary Figs. 15 and 16). The effect of using an improved drinking water source on child diarrhoea also did not meaningfully change when variables describing drinking water contamination at the POC and drinking water accessibility were removed from the main subcomponent model (RR 0.99, 95% CI 0.91–1.09). Results from a household-clustered analysis including all children (*n* = 40,407) were broadly consistent with the primary findings; however, estimates for two subcriteria shifted: accessibility on premises showed a stronger protective association and improved drinking water source type shifted slightly above the null with borderline evidence (Supplementary Table 5). These differences reflect the shift from a household-weighted analysis (one child per household) to a child-weighted analysis (all children included), and suggest greater uncertainty in the estimated roles of accessibility and water source type across household compositions.

## Discussion

Multiple studies show the benefits of improving access to safe water on child health. However, there remains a paucity of data on the impact of achieving universal safe drinking water access based on the UN’s SDG framework on child diarrhoeal disease^16^. Analysing household survey data from 24 countries, we observed a significant protective effect of the use of SMDWS compared with the use of drinking water services not fulfilling the SDG 6.1 criteria on diarrhoea in children under 5 years. When analysing the independent effects of the subcriteria used to define SMDWS, availability of drinking water when needed had the largest risk reduction on childhood diarrhoeal disease. Use of drinking water with no detectable faecal contamination at the POU was identified as the household drinking water service dimension that contributed to the largest reduction in childhood diarrhoeal cases in our study population but is notably not a part of the SMDWS criteria (which includes POC contamination). Defining SMDWS by absence of faecal contamination at the POU rather than the POC, however, did not substantially reduce RRs of child diarrhoea. We also observed that climate zones and weather conditions influence the effectiveness of SMDWS use in reducing diarrhoea, which may help to explain the heterogeneity observed in controlled trials.

Compared with trial-based estimates underpinning burden of disease models, we find more modest diarrhoea reductions associated with SMDWS across study populations from 24 countries^15,16^. Estimates of the global burden of diarrhoeal disease attributable to unsafe drinking water have used pooled RR estimates from two controlled trials of higher drinking water service levels, one in Bangladesh and one in Puerto Rico^15,49,50^. Our estimates of diarrhoeal risk reductions associated with the use of SMDWS compared with the use of less than basic drinking water services (RR 0.87, 95% CI 0.78–0.97) are substantially lower than risk reductions associated with higher drinking water service levels compared with the use of unimproved services derived from these controlled trials (RR 0.48, 95% CI 0.26–0.87)^16^. Higher risk reductions derived from the respective trials may be influenced by additional benefits of the interventions (for example, educational campaigns and repeated visits from health care workers), social desirability bias or other factors specific to the geographic setting^51,52^. Our results, on the other hand, reflect an average effect from cross-sectional data for 24 countries. Our lower estimate of risk reduction may be influenced by differences in the definition of exposure and outcome variables to those used in controlled trials and unmeasured confounding variables in our analysis^53^. Nevertheless, consistency in the findings of overall benefits of higher drinking water service levels on diarrhoeal outcomes from controlled trials and with observational analyses from a broad range of household surveys provides increased external validity on the effects of SMDWS on child health in LMICs^54^.

Our findings suggest that achieving SDG target 6.1, as currently defined, may yield smaller reductions in the childhood diarrhoeal disease burden than previously estimated. This is in part because the SDG indicator focuses on water quality at the POC, while the disease burden estimate considers contamination at the POU. The focus of SDG target indicator 6.1.1 on POC contamination reflects the general perception that absence of contamination at the POC is the responsibility of the water provider whereas absence of contamination at the POU is the responsibility of the household^14^. Providing universal access to SMDWS was estimated to prevent 35% (95% CI 31–40%) of the diarrhoeal disease burden in 132 LMICs in 2019 when contamination at the POU is considered^15^. We estimate that a substantially lower fraction of diarrhoeal disease cases would be prevented by fulfilling the SMDWS criteria as defined for SDG indicator 6.1.1 (6.8%, 95% CI 1–11%), which only considers contamination at the POC, across the 24 LMICs in our study population. Although we observed no significant difference in the RR of diarrhoea associated with SMDWS use defined using water quality at the POC versus POU, substantially fewer households in LMICs meet SMDWS criteria when POU contamination is considered. We note that water contamination estimates are only one source of uncertainty in estimates of the global diarrhoeal disease burden attributable to unsafe drinking water: modelled SMDWS coverage is highly uncertain in settings where nationally representative household water quality data are lacking.

To understand which dimensions of SMDWS are the most important for reducing diarrhoeal disease, we examined effects of the SDG 6.1 criteria on the RR of child diarrhoea separately. We found that water availability when needed showed the greatest protective associations with child diarrhoea. Water insufficiency has been reported to increase risks of diarrhoeal disease by limiting water for hand hygiene practices^55^, increasing reliance on alternative unsafe water sources and storage practices^56,57^. If intermittency in piped supplies is the cause of limited water availability, risks may also be exacerbated by the potential for intrusion and backflow of faecal contamination during periods of low pressure^58^. By contrast, improving drinking water contamination at POC and water source type were not associated with reduced child diarrhoea risks in our study population. Drinking water contamination at the POU, which is not part of SDG 6.1.1, was significantly and substantially associated with reduced diarrhoeal risks. Our sensitivity analysis also pointed towards the protective effect of accessibility on premises when multiple children per household were included in the model, highlighting uncertainties and possible heterogeneity in effect sized across households of different sizes. Although our results may be representative of general trends across geographies represented in our study, we highlight that the relative importance of drinking water service dimensions will probably vary by context.

Our results underline the importance of improving household drinking water quality at the POU for child health and provide new evidence on the relationship between *E. coli* counts in stored drinking water and child diarrhoeal disease. Despite the broad use of *E. coli* as a faecal indicator, there is limited and conflicting quantitative evidence on the relationship of *E. coli* concentrations in drinking water and child diarrhoea. Some studies report that moderate increases in household drinking water contamination are associated with increased risks of diarrhoea while others report that only high concentrations, for example, above 1000 *E. coli* CFU per 100 ml, show substantial effects^45,59^. Our study shows incremental increases in diarrhoeal disease risk with each WHO *E. coli* risk category at the POU but not at the POC. Household drinking water storage is a major risk factor for faecal contamination because unhygienic storage containers, extraction utensils and direct hand contact with water can contaminate water supplies^36,60,61^. We found that among households classified as having SMDWS at the POC, the POU water was contaminated in approximately one out of three cases. Given that billions of people are exposed to faecal contamination through their drinking water globally^12^, the WHO risk categories at the POU provide relevant information for identifying priority areas. To achieve greater reductions in diarrhoeal disease than would be expected by achieving SDG 6.1, interventions that go beyond drinking water quality at the POC and radically limit pathogen transmission in domestic environments via additional routes such as soil, food, hands and flies are probably needed^4,51^.

Our results underline the role of climate in the relationship between SMDWS and diarrhoea. Effects of SMDWS use on diarrhoeal disease were more pronounced in children residing in tropical regions compared with arid or temperate regions. Tropical climate zones are typically characterized by pronounced seasonality, and prevalence of diarrhoeal pathogens such as rotavirus has been reported to be affected by weather in tropical regions^5,6^. Our results suggest that use of SMDWS may be less effective during transitions from dry to wet periods, potentially due to concentration–dilution effects^20^. These results complement findings from a meta-data analysis of 34 controlled drinking water intervention trials, which showed stronger effects on diarrhoea in the dry season than in the wet season^62^. An alternative interpretation is that climate and weather impacts on diarrhoea are modified by the use of SMDWS^7^. Further research is needed to better understand the causal mechanisms and to explain heterogeneity in effect sizes across climate zones and rainfall patterns.

There are several limitations to our study, which are mainly linked to data quality, availability and structure. Owing to limited data availability of SMDWS and child diarrhoea, we could not equally represent all UN regions and climate zones, with limited representation of LMICs in the Americas and cold climates and no LMICs representing contexts in Europe or Oceania in our dataset. Missing data on child diarrhoea for 35,058 out of 77,873 households with nationally representative information on SMDWS also means that our data may not be nationally representative of the included countries. Our outcome indicator is caregiver-reported diarrhoea in the past 2 weeks as this is the indicator collected by the MICS survey. Caregiver-reported diarrhoea is prone to subjectivity and recall bias, which contributes to a demonstrated low sensitivity and potential underestimation of diarrhoeal prevalence^63^. Notably, the indicator for faecal contamination in the MICS surveys is the presence of *E. coli* in drinking water, which is widely used owing to the ready availability of field-portable detection methods, relative low cost and ease of use. Nevertheless, it is important to note that *E. coli* is an imperfect indicator. First, the presence of *E. coli* is not necessarily indicative of the presence of diarrhoeal pathogens owing to differences in potential sources (people, birds and animals), transport processes and environmental persistence. Furthermore, household drinking water quality, as indicated by *E. coli*, can vary rapidly^64^. Given that household surveys assess water quality at only one timepoint, the results may not necessarily be representative of children’s previous faecal exposures. Notably, data on chemical contamination was not included owing to lack of data availability but may also affect diarrhoea outcomes^65^. Finally, our observational analysis could be affected by unmeasured confounding variables and is limited by the lack of longitudinal data on SMDWS.

## Methods

### Structural causal models

In this study, we adopted a causal modelling approach to analyse observational data on household drinking water services and childhood diarrhoea as hypothetical controlled trials. We built structural causal models represented as DAGs to explicitly describe our causal assumptions on all exposures and confounding factors relevant for estimating the total effect of SMDWS interventions as well as interventions on drinking water source type, accessibility, availability and quality on diarrhoea in children under 5 years^66^. Exposure routes for all DAGs were defined based on literature (Supplementary Table 1) and expert opinion, and minimal sufficient adjustment sets of variables required to quantify the total effects of drinking water services on child diarrhoea were identified using the Daggity (v3.1) software^24^. For our primary analysis, we created two DAGs to estimate the total effect of SMDWS, defined at the POC on childhood diarrhoea in LMICs (Supplementary Figs. 1 and2). For our secondary analysis a third DAG was developed to estimate the total effect of the individual subcomponent criteria of SMDWS (the use of a drinking water source that is (1) improved, (2) accessible, (3) available when needed and (4) free of faecal contamination at the POC) on childhood diarrhoea (Supplementary Fig. 3). A fourth DAG was developed to estimate the total effect of faecal contamination at the POU on childhood diarrhoea (Supplementary Fig. 4). An overview of all models and variables included in the primary and secondary analysis is given in Supplementary Table 4.

### Data

We used nationally representative household and child health survey data gathered within the framework of the UNICEF MICS Programme between 2016 and 2020 from LMICs. Twenty-six household surveys were selected based on the availability of information on diarrhoea period prevalence in children under 5 years, drinking water quality at the POC, drinking water quality at the POU, drinking water source type, accessibility and availability. Data were downloaded from the MICS website as SPSS files and processed in R studio (version 2024.12.1 + 563), using the R programming language (version 4.2.2) and R packages haven (version 2.5.2) and tidyverse (version 2.0.0)^40,67–70^. Before merging the household and child’s health survey data files, all the relevant variables were checked to ensure the naming of variables and categories were consistent or were otherwise adjusted. Of the 527,402 observations of children under 5 years included in these surveys, 485,719 were excluded due to lack of data on diarrhoea period prevalence, drinking water quality at the POC or POU, drinking water source type, accessibility or availability. Furthermore, 264 observations were excluded due to lack of information on child age, mother level of education or because they lived in a camp rather than a rural or urban area. We selected one randomly chosen child per household, excluding 13,866 children who were living in a household with another child under 5 years to avoid intrahousehold correlation. Finally, we excluded 765 observations due to lack of information on total precipitation or average surface temperature in the area the surveys were taken. This resulted in a final dataset for our analysis that included 26,796 observations of children under 5 years from 24 LMICs (Supplementary Fig. 17).

MICS household survey question responses were used to create indicators for SMDWS use as well as its subcomponents at household level according to the JMP criteria^11^. The indicator used to monitor SDG 6.1 includes data on the presence or absence of *E. coli* in a 100 ml sample at the POC. When the MICS surveys assess *E. coli* contamination, they record, in addition to presence or absence, the concentration of *E. coli* in a sample as CFU in a 100 ml sample at the POC and POU. SMDWS were defined in two different ways: (1) using drinking water quality data on *E. coli* prevalence in a 100-ml drinking water sample at POC from the household’s primary drinking water source (note that this is the way SMDWS are defined for monitoring SDG indicator 6.1.1) or (2) using data on *E. coli* prevalence in a 100-ml stored drinking water sample from the POU (note that this definition is used for global burden of disease calculations). Furthermore, *E. coli* risk categories of low (<1 CFU per 100 ml), intermediate (1 < 10 CFU per 100 ml), high (10 < 100 CFU per 100 ml) and very high (>100 CFU per 100 ml) levels of contamination, for both POC and point of use were additionally defined according to the WHO drinking water quality guidelines^14^.

To match environmental data with the household survey data, we used the region names reported in the household surveys to identify the global administrative (GADM) area within which the households were located. In some cases, the regions specified in the survey data did not directly correspond to a predefined GADM region, in which case we defined combined GADM regions of lower administrative levels to identify district boundaries of the surveyed area. We sampled total precipitation and average temperature of the Earth’s surface over 30 days before the corresponding survey date, averaged across each corresponding administrative district using the web-based code editor in Google Earth Engine (GEE)^43^. The CHIRPS Precipitation Daily: Climate Hazards Center InfraRed Precipitation With Station Data (version 2.0 Final) dataset was used for precipitation indicators and the ERA5–Land Daily Aggregated–ECMWF Climate Reanalysis data for temperature indicators^41,42^.

### Statistical analysis

We used log-binomial generalized linear mixed models with a random intercept for each country *j* to estimate the RR of a child *i* having diarrhoea in the previous 2 weeks living in a household which used SMDWS at the POC (model 1.1) or SMDWS at the point of use (model 1.2) (R package lme4, version 1.1–33)^71^. We assumed a binomial distribution of our data. The expected outcome value is modelled as an exponential function of the linear predictors using a log-link function. In a secondary analysis, we used the same model structure but substituted the SMDWS variables in model 1.1 and model 1.2 with indicators for drinking water availability, accessibility on premises, drinking water source type and faecal contamination at the POC (model 2.1) or POU (model 2.2). An overview of equations with detailed descriptions of the included variables are described below.

### Main models

Model 1.1

$$\log \left({\mathbb{E}}\left[{\mathrm{diarrhoea}}_{ij}\right]\right)={\beta }_{0}+{\beta }_{1}\,{\mathrm{SMDWS}}_{ij}$$

$$+{\beta }_{2}\,{\mathrm{Age}\,\mathrm{group}}_{{ij}}+{\beta }_{3}\,{\mathrm{Sex}}_{{ij}}+{\beta }_{4}\,{\mathrm{Mother}\,\mathrm{education}\,\mathrm{level}}_{{ij}}$$

$$\begin{array}{l}+{\beta }_{5}\,{\mathrm{Wealth}\,\mathrm{quintile}}_{ij}+{\beta }_{6}\,{\mathrm{Area}}_{ij}+{\beta }_{7}\,{\mathrm{Basic}\,\mathrm{sanitation}}_{ij}\\ +{\beta }_{8}\,{\mathrm{Total}\,\mathrm{precipitation}}_{ij}+{\beta }_{9}\,{\mathrm{Mean}\,\mathrm{temperature}}_{ij}+{\upsilon }_{j}.\end{array}$$

Model 1.2

$$\log \left({\mathbb{E}}\left[{\mathrm{diarrhoea}}_{ij}\right]\right)={\beta }_{0}+{\beta }_{1}\,{\mathrm{SMDWS}\,\mathrm{at}\,\mathrm{POU}}_{ij}$$

$$+{\beta }_{2}\,{\mathrm{Age}\,\mathrm{group}}_{{ij}}+{\beta }_{3}\,{\mathrm{Sex}}_{{ij}}+{\beta }_{4}\,\mathrm{Mother}\,\mathrm{education}\,{\mathrm{level}}_{ij}$$

$$\begin{array}{l}+{\beta }_{5}{{\mathrm{Wealth}}\,{\mathrm{quintile}}}_{ij}+{\beta }_{6}{\mathrm{Area}}_{ij}+{\beta }_{7}{{\mathrm{Basic}}\,{\mathrm{sanitation}}}_{ij}\\ +{\beta }_{8}{{\mathrm{Basic}}\,{\mathrm{hygiene}}}_{ij}+{\beta }_{9}{{\mathrm{Total}}\,{\mathrm{precipitation}}}_{ij}\\+{\beta }_{10}{{\mathrm{Mean}}\,{\mathrm{temperature}}}_{ij}+{\upsilon }_{j}\end{array}$$

where *β*_0_ is the overall intercept, *β*_1_ is the fixed effect of SMDWS use (exposure variable), *β*_2_–*β*_10_ are the fixed effects of control variables and *υ*_*j*_ is the random intercept at country level, assumed to be independent across countries and normally distributed.

Diarrhoea is defined as a binary outcome based on the survey response of caregivers on whether the child had diarrhoea (‘1’) or not (‘0’) within the previous 2 weeks.

SMDWS is either

- a binary exposure variable (‘Yes’/‘No’ (reference)) and represents a household’s use of SMDWS, which is defined through the fulfilment of the following criteria based on JMP methodology^11^:
  1. Primary drinking water source is classified as improved
  2. The primary or secondary drinking water source is accessible on premises
  3. Household members had not experienced drinking water insufficiency within the previous month
  4. A 100 ml drinking water sample taken at the POC was free of contamination, using *E. coli* as a faecal indicator
- Or a categorical exposure variable with three categories:
  1. Using SMDWS
  2. Using services which are at least basic but not SMDWS
  3. Using services which are not basic
  4. Households fall within the ‘at least basic but not SMDWS’ category if they use drinking water services, which are at least basic but do not fulfil all the four SMDWS criteria. They fall within the ‘not basic’ category if they do not fulfil the criteria of basic drinking water service use according to the JMP methodology, which require that:
  5. The primary drinking water source is improved
  6. The time it takes to fetch water is not more than a 30-min round trip including queuing.

SMDWS at POU differs from SMDWS only in the way that the drinking water sample for criteria (4) is taken from the point of use rather than the POC. It is used as a binary or categorical variable as described above for SMDWS.

Age group is a categorical control variable, with five age groups (‘<12 months’ (reference group)/‘12–24 months’/‘24–36 months’/‘36–48 months’/‘48–60 months’).

Sex is as a binary control variable (‘Female’ (reference)/‘Male’).

Mother education level is a binary control variable (‘Primary or no education’ (reference)/‘Secondary or higher education’). Notably, this classification may differ across countries based on national school systems.

Wealth quintile is an ordinal control variable which represents a household’s living standards. It is calculated individually for each country using a unique set of assets owned by each household. Households are categorized from poorest (windex51 (reference)) to richest (windex55).

Area is a binary control variable (rural (reference)/urban). Basic sanitation is a binary control variable (‘Yes’/‘No’ (reference)), which was defined based on the JMP sanitation service ladder. It is defined through access to a sanitation facility which is improved and not shared.

Basic hygiene is a binary control variable (‘Yes’/‘No’ (reference)), which was defined based on the JMP hygiene service ladder. It is defined through the observation of a handwashing facility on premises as well as availability of water and soap.

Total precipitation is a continuous control variable in decimetres and is representative of total precipitation over 30 days before survey data collection across the administrative district the survey was collected in.

Mean temperature is a continuous control variable in degrees Celsius and is representative of the mean temperature of the Earth’s uppermost surface layer over 30 days before survey data collection across the administrative district the survey was collected in.

### Secondary analysis of SMDWS criteria

Model 2.1

$$\log \left({\mathbb{E}}\left[{\rm{diarrhoea}}_{ij}\right]\right)={\beta }_{0}+{\beta }_{1}\, {\rm{Improved}}\,{\rm{source}}_{ij}+{\beta }_{2} \,{\rm{Accessible}}$$

$$+{\beta }_{3}\,\mathrm{Available}\,\mathrm{when}\,\mathrm{needed}+{\beta }_{4}\,\mathrm{Faecal}\,\mathrm{contamination}\,\mathrm{at}\,\mathrm{POC}+$$

$$+{\beta }_{5}\,{\mathrm{Age}\,\mathrm{group}}_{{ij}}+{\beta }_{6}\,{\mathrm{Sex}}_{{ij}}+{\beta }_{7}\,{\mathrm{Mother}\,\mathrm{education}\,\mathrm{level}}_{{ij}}$$

$$\begin{array}{l}+{\beta }_{8}\,{\mathrm{Wealth}\,\mathrm{quintile}}_{ij}+{\beta }_{9}\,{\mathrm{Area}}_{ij}+{\beta }_{10}\,{\mathrm{Basic}\,\mathrm{sanitation}}_{ij}\\ +{\beta }_{11}\,{\mathrm{Total}\,\mathrm{precipitation}}_{ij}+{\beta }_{12}\,{\mathrm{Mean}\,\mathrm{temperature}}_{ij}+{\upsilon }_{j}.\end{array}$$

Model 2.2

$$\log \left({\mathbb{E}}\left[{\mathrm{diarrhoea}}_{ij}\right]\right)={\beta }_{0}+{\beta }_{1}\,{\mathrm{Improved}\,\mathrm{source}}_{ij}+{\beta }_{2}\,\mathrm{Accessibl}{\bf{e}}$$

$$+{\beta }_{3}\,\mathrm{Available}\,\mathrm{when}\,\mathrm{needed}+{\beta }_{4}\,\mathrm{Faecal}\,\mathrm{contamination}\,\mathrm{at}\,\mathrm{POU}+$$

$$+{\beta }_{5}\,{\mathrm{Age}\,\mathrm{group}}_{{ij}}+{\beta }_{6}\,{\mathrm{Sex}}_{{ij}}+{\beta }_{7}\,{\mathrm{Mother}\,\mathrm{education}\,\mathrm{level}}_{{ij}}$$

$$\begin{array}{l}+{\beta }_{8}\,{\mathrm{Wealth}\,\mathrm{quintile}}_{ij}+{\beta }_{9}\,\mathrm{Are}{a}_{ij}+{\beta }_{10}\,{\mathrm{Basic}\,\mathrm{sanitation}}_{ij}\\ +{\beta }_{11}{\,\mathrm{Basic}\,\mathrm{hygiene}}_{ij}+{\beta }_{12}\,{\mathrm{Total}\,\mathrm{precipitation}}_{ij}\\+{\beta }_{13}\,{\mathrm{Mean}\,\mathrm{temperature}}_{ij}+{\upsilon }_{j,}\end{array}$$

where *β*_0_ is the overall intercept, *β*_1_–*β*_4_ are the fixed effect of exposure variables, *β*_5_–*β*_13_ are the fixed effects of control variables and *υ*_*j*_ is the random intercept at country level, assumed to be independent across countries and normally distributed.

Improved source is either

- a binary exposure variable (‘Yes’/‘No’ (reference)) and describes whether a household uses an improved primary drinking water source based on the JMP classification of improved sources^11^
- or it is a categorical exposure variable (‘unimproved’ (reference)/‘improved but not piped’/‘piped’).

Accessible is a binary exposure variable (‘Yes’/‘No’ (reference)) and describes whether a household uses a primary or secondary drinking water source which is within the dwelling or in the yard or plot.

Available when needed is a binary exposure variable (‘Yes’/‘No’ (reference)) and describes whether household members experienced drinking water insufficiency in the previous month.

Faecal contamination at POC or POU are either

- binary exposure variables (‘Yes’/‘No’ (reference))
- or categorical risk categories (‘low’ (<1 CFU per 100 ml) (reference)/‘intermediate’ (1 < 10 CFU per 100 ml)/‘high’ (10 < 100 CFU per 100 ml)/‘very high’ (>100 CFU per 100 ml)).

### Calculating the population attributable fraction of disease

The population attributable fraction (PAF) is a standard epidemiological method to quantify disease burden which could have been prevented by removing a specific risk factor. In our case, we quantify the number of diarrhoeal cases in children under 5 years that could have been prevented by providing safe drinking water services. We calculate this with the following equation:

$$\mathrm{PAF}=\frac{p\, \,(1-{\mathrm{RR}})}{1+p\, \,(1-{\mathrm{RR}})},$$

where *p* is the fraction of the population using unsafe drinking water services and RR is the RR reduction associated with using safe drinking water services.

### Subgroup analysis by climatic conditions

We subgrouped our survey data based on which Köppen–Geiger climate zone the households were located in as well as whether the surveys were gathered within 30 days after the onset of a wet period. For this, we overlaid a high-resolution map of the five main Köppen–Geiger climate zones (arid, cold, temperate, tropical and polar) from 1991 to 2020 with the surveyed GADM areas in GEE^44^. We sampled pentad (5 day) total precipitation for six pentads before and two pentads after each individual survey date in GEE. We determined whether the survey had been gathered within 30 days of the onset of a wet period which lasted at least 15 days based on a simplified version of the ‘anomalous accumulation’ method by Liebmann et al.^72^. For this, we calculated the anomalous accumulation for six consecutive pentads before and two after the survey date as

$$A\left(\mathrm{pentad}\right)=\mathop{\sum }\limits_{n=1}^{\mathrm{pentad}}\left[R\left(n\right)-\bar{R}\right],$$

where *R*(*n*) is total pentad precipitation and *R* is the climatological annual pentad average based in our case on pentad precipitation sampled over a period of 305 days before the sample date and 60 days after. A survey is classified as being within 30 days of the onset of a wet period lasting at least 15 days if the anomalous accumulation remains positive for three consecutive pentads starting from one to six pentads before the survey date (Supplementary Fig. 18).

### Sensitivity analysis

We tested the robustness of our results in several ways investigating impacts of finer-scale clustering, random sampling of one observation per household and collinearity. This involved refitting the primary SMDWS and subcomponent models to include a subnational district-level random intercept nested within countries to account for subnational spatial clustering. To investigate the effect of the randomly selecting one child per household we performed a Monte Carlo resampling procedure: we randomly selected one child per household and refit the model 100 times, summarizing the median effect estimate and 95% confidence limits across resamples. In both analyses, minimal covariate adjustment sets based on DAGs presented in Supplementary Figs. 11 and 13 were controlled for. We additionally fit a marginal model using the same covariate set as the primary analysis and included all eligible children (*n* = 40,407), using household-clustered robust standard errors to account for intrahousehold correlation (R package clubsandwich, version 0.6.1^73^). Finally, we investigated collinearity of the SMDWS subcomponents by measuring tetrachoric correlations for binary covariates (Supplementary Table 6). We removed highly correlated exposures from the subcomponent analysis and reported changes in RRs and CIs of childhood diarrhoea. Specifically, we removed the variable describing water source type from the main model because it was highly correlated with drinking water contamination at the POC (tetrachoric correlation coefficient (tcc) of −0.7), POU (tcc of −0.55) and accessibility on premises (tcc of 0.64) (Supplementary Table 6). We also modelled the subcomponents water source type and drinking water availability, while removing drinking water accessibility and *E. coli* contamination at the POC.

## Supplementary information

Supplementary Information Supplementary Figs. 1–18 and Tables 1–7.

Supplementary Data 1 Supplementary dataset containing additional descriptive statistics on the combined datasets including population demographics for the main model, for a sensitivity analysis, and stratified by country, climate zone and wet/dry weather period.

## Data Availability

Data used in this study were extracted from the following sources: household survey variables were taken from the Multiple Indicator Cluster Survey data and are provided by UNICEF through the MICS website upon request and under specified use conditions^40^. Data sources of climate variables are cited in the Methods under author-specified Creative Commons licences.

## Code availability

The code needed to replicate the analysis described in this study is available via Zenodo and GitHub under a MIT license at https://doi.org/10.5281/zenodo.19102224 (ref. ^74^) and https://github.com/EawagPHH/effect-of-SMDWS-on-child-diarrhoea/tree/v1.0.0 (ref. ^75^).
