# Supplementary material for: Safely managed drinking water service use and child diarrhoea based on evidence from 24 countries
Source: Nat Water. 2026 Jun 2;4(6):764–75. doi: 10.1038/s44221-026-00647-4 (PMC13652726; doi:10.1038/s44221-026-00647-4)
Supplement: Supplementary file 1 [file 44221_2026_647_MOESM1_ESM.pdf]

nature water

Supplementary information https://doi.org/10.1038/s44221-026-00647-4

Safely managed drinking water service use and child diarrhoea based on evidence from 24 countries

In the format provided by the authors and unedited

Table of Contents

Figure S1 3

Figure S2 4

Figure S3 5

Figure S4 6

Figure S5 7

Figure S6 8

Figure S7 9

Figure S8 10

Figure S9 11

Figure S10 12

Figure S11 13

Figure S12 14

Figure S13 15

Figure S14 16

Figure S15 17

Figure S16 18

Figure S17 19

Figure S18 19

Table S1 20

Table S2 22

Table S3 23

Table S4 24

Table S5 25

Table S6 26

Table S7 27

Other Supplementary Materials for this manuscript include:

Tables S1

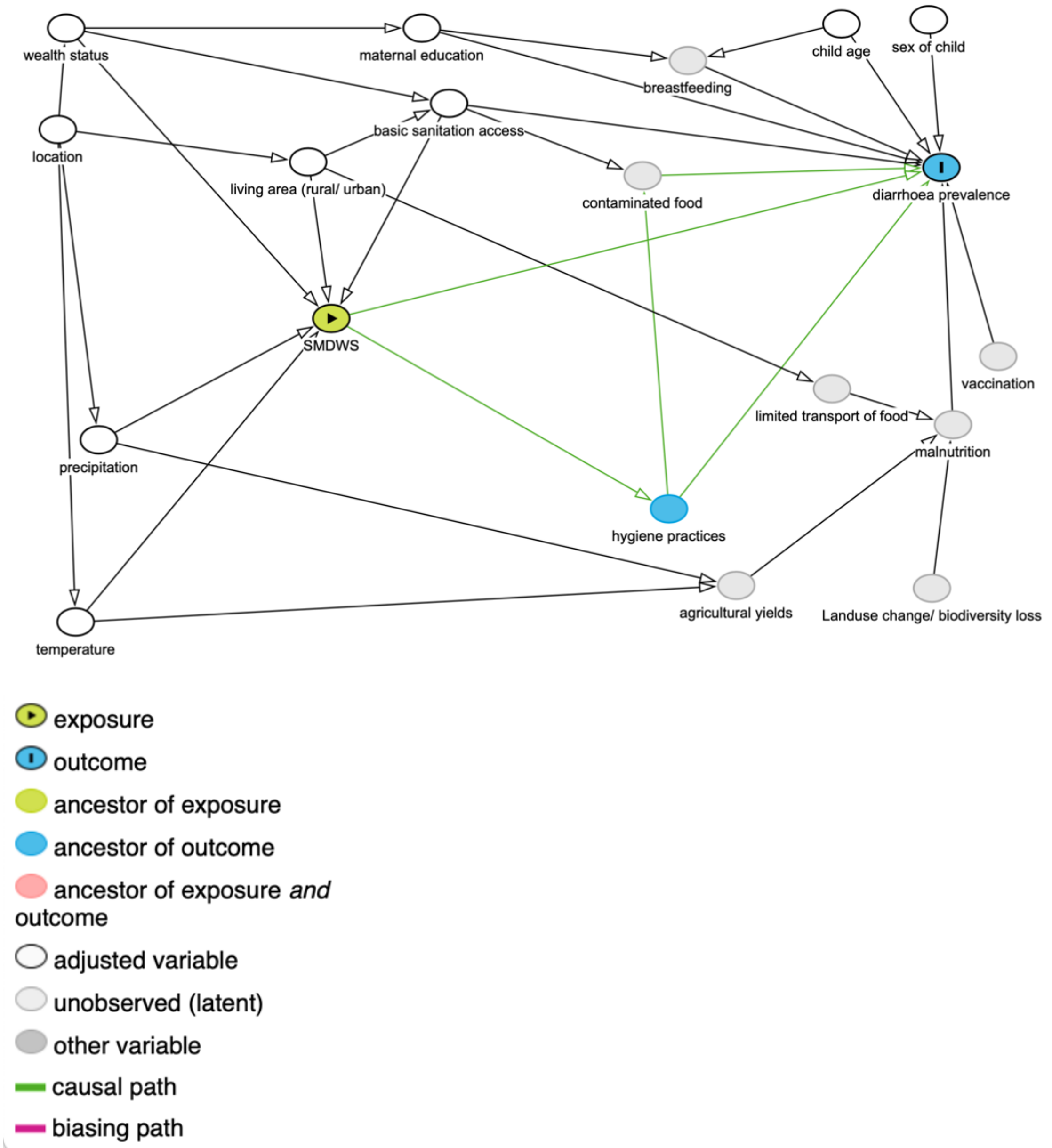

Figure S1**:** Correctly adjusted Directed Acyclic Graph (DAG) for estimating the total effect of the safely managed drinking water services (SMDWS) defined using drinking water quality at the point of collection (POC) on child diarrhoea prevalence with adjustment sets containing basic sanitation access, child age, living area (rural/ urban), location, maternal education, precipitation, sex of child, temperature, wealth status. Accessed on 11 September 2024: https://dagitty.net/m8oHXPBBr

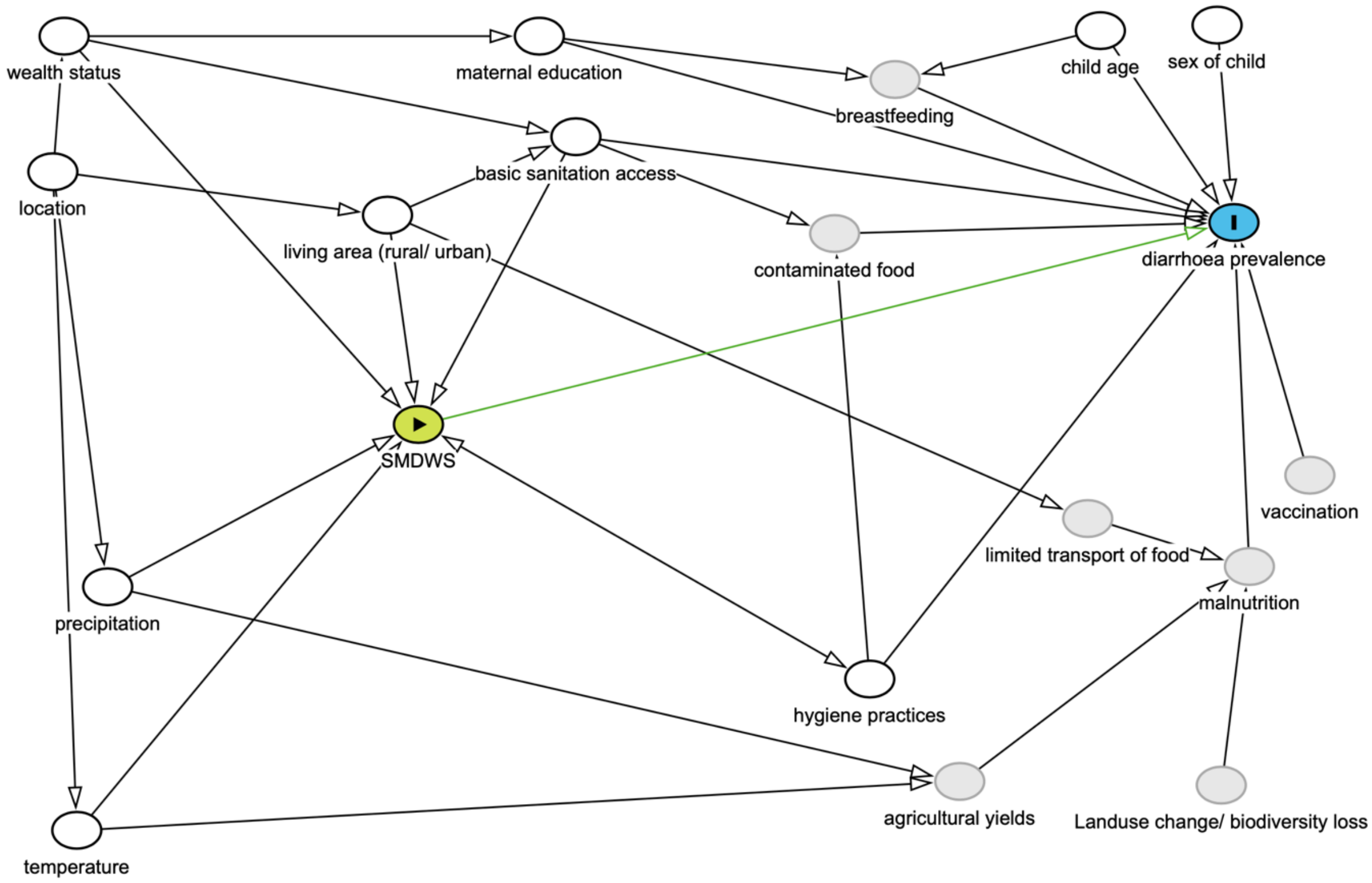

Figure S2**:** Correctly adjusted DAG for estimating the total effect of the SMDWS defined at the point of use (POU) on child diarrhoea prevalence with adjustment sets containing basic sanitation access, child age, hygiene practices, living area (rural/ urban), location, maternal education, precipitation, sex of child, temperature, wealth status

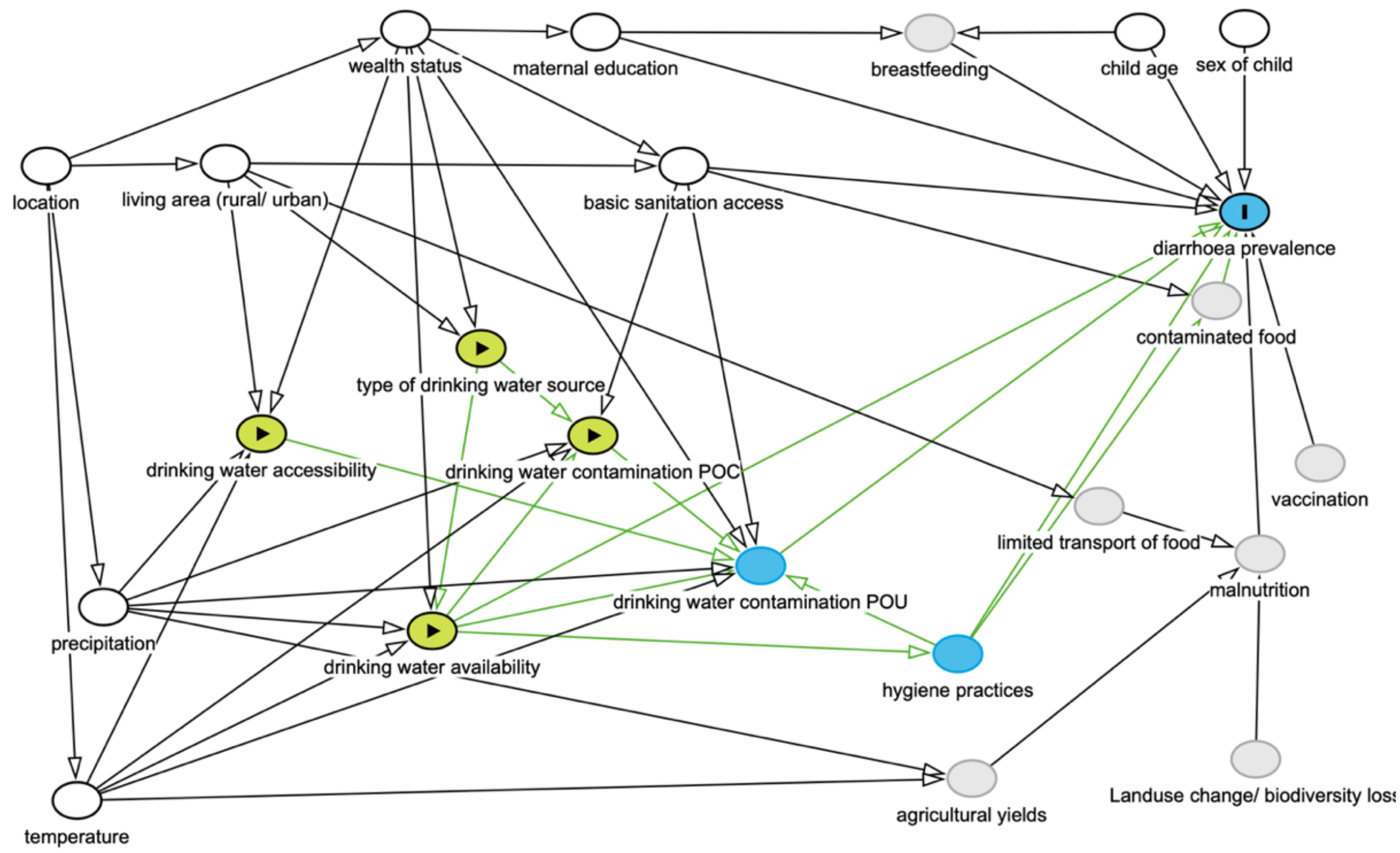

Figure S3**:** Correctly adjusted DAG for estimating the total effect of the SMDWS subcomponents (drinking water source type, availability, accessibility and faecal contamination at the POC) on diarrhoea prevalence with adjustment sets containing basic sanitation access, living area (rural/ urban), location, maternal education, child age, sex of child, precipitation, temperature, wealth status. Accessed on 11 September 2024: https://dagitty.net/mXYisDzoc

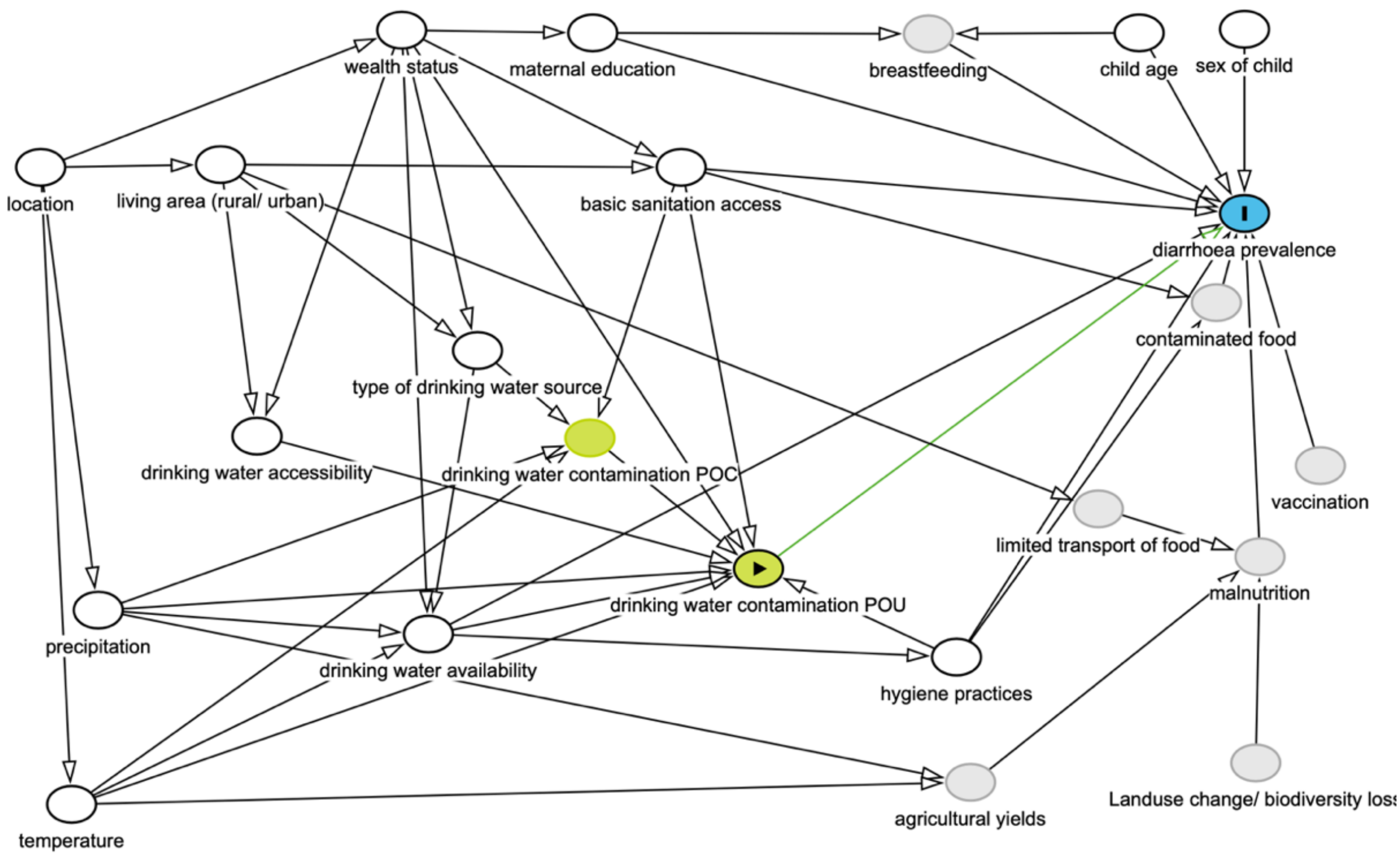

Figure S4**:** Correctly adjusted DAG for estimating the total effect faecal contamination at the POU on diarrhoea prevalence with adjustment sets containing basic sanitation access, hygiene practices, living area (rural/ urban), location, maternal education, child age, sex of child, precipitation, temperature, wealth status.

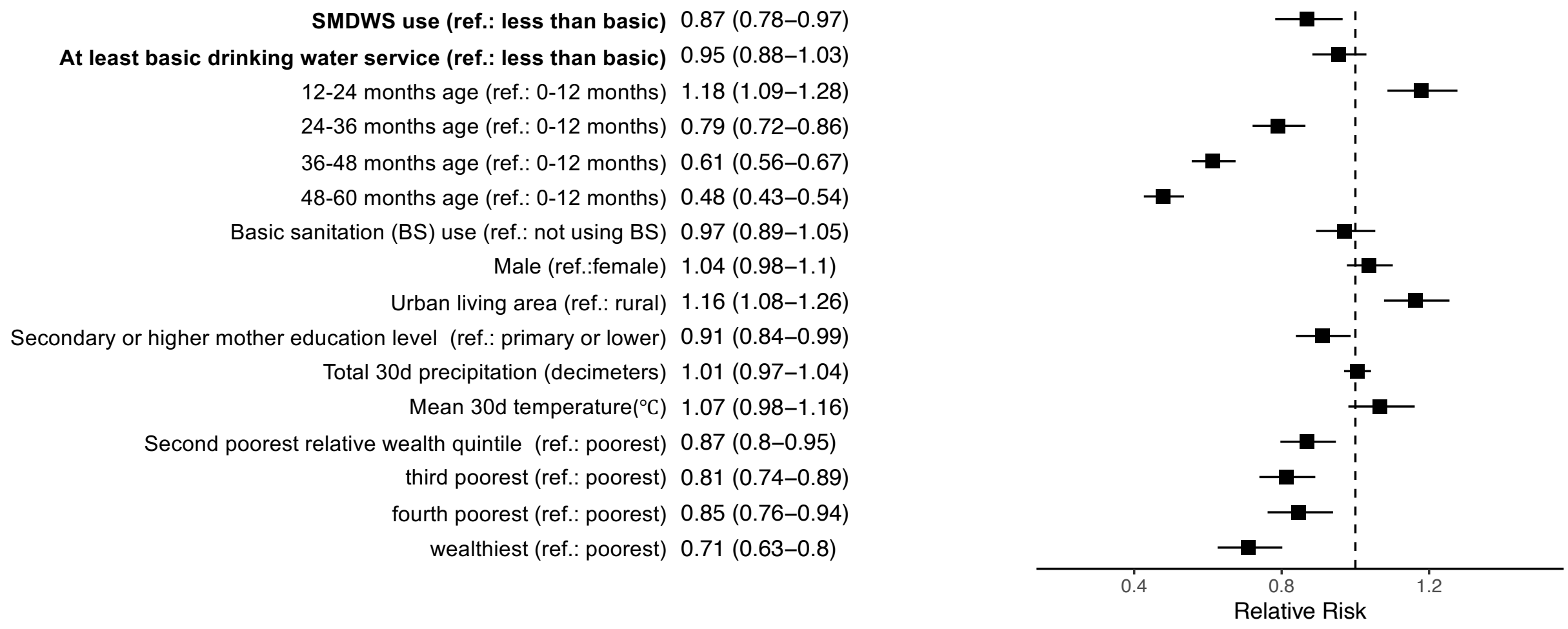

Figure S5**:** Adjusted relative risk for diarrhoeal disease in children living in households using SMDWS at the point of collection (n=7320) and using drinking water services which are at least basic but not safely managed (n=11449) compared to those in households using services which are less than basic (n=8027). Squares represent point estimates of relative risk and horizontal lines the 95% confidence intervals including those of adjusted variables. The dashed vertical at relative risk = 1 marks no change of risk.

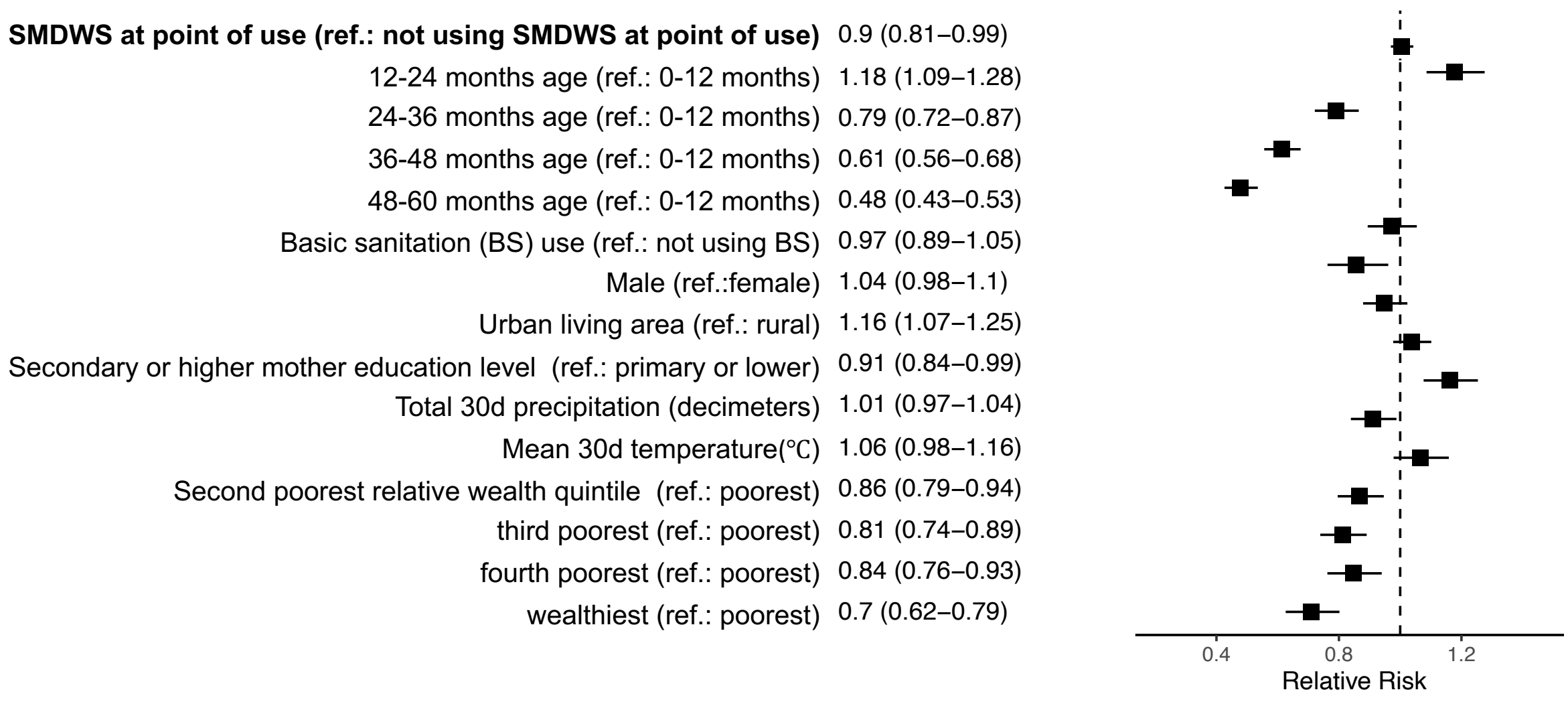

Figure S6**:** Adjusted relative risk for diarrhoeal disease in children living in households using SMDWS at the POU (n=5204) compared to households not fulfilling all of the SMDWS criteria at the POU (n=21592). Relative risk of adjusted variables is also reported. Squares represent point estimates of relative risk and horizontal lines the 95% confidence intervals including those of adjusted variables. The dashed vertical at relative risk = 1 marks no change of risk.

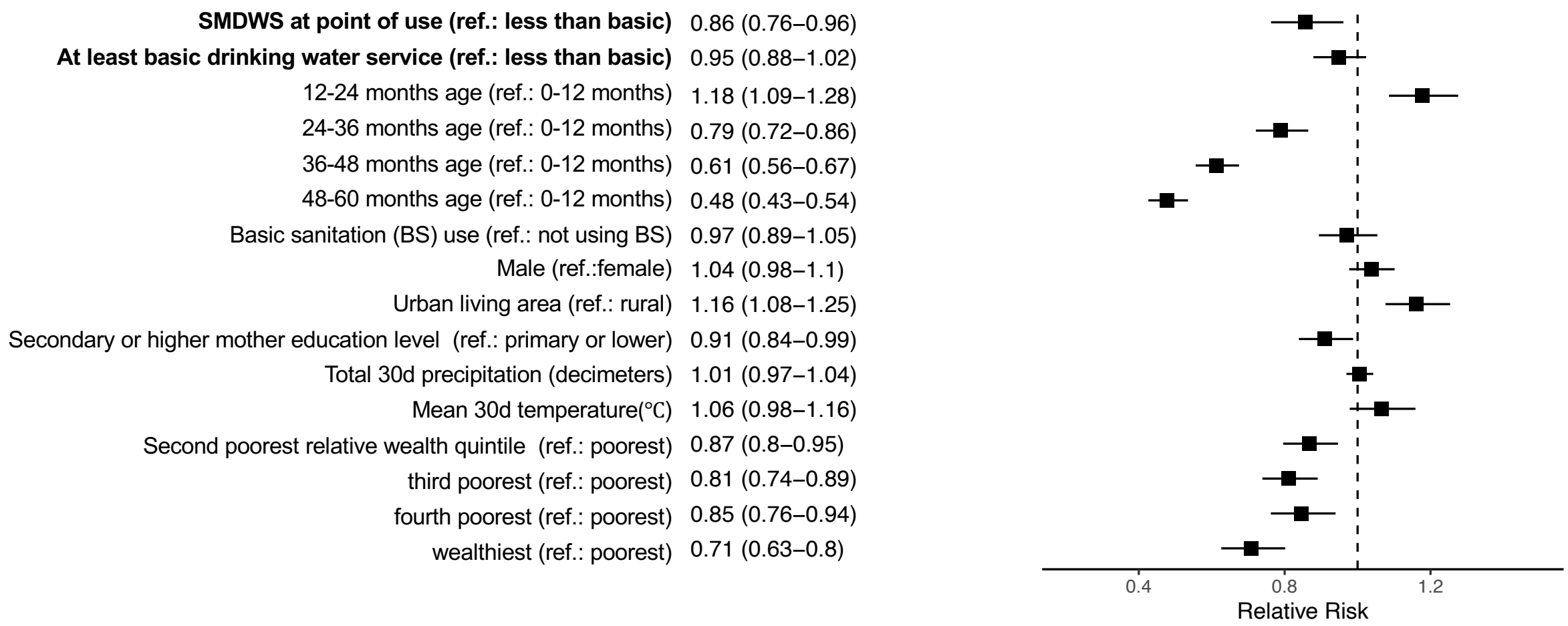

Figure S7**:** Adjusted relative risk for diarrhoeal disease in children living in households using SMDWS at the POU (n=5204) and using drinking water services which are at least basic but not safely managed (n=13565) compared to those in households using services which are less than basic (n=8027). Squares represent point estimates of relative risk and horizontal lines the 95% confidence intervals including those of adjusted variables. The dashed vertical at relative risk = 1 marks no change of risk.

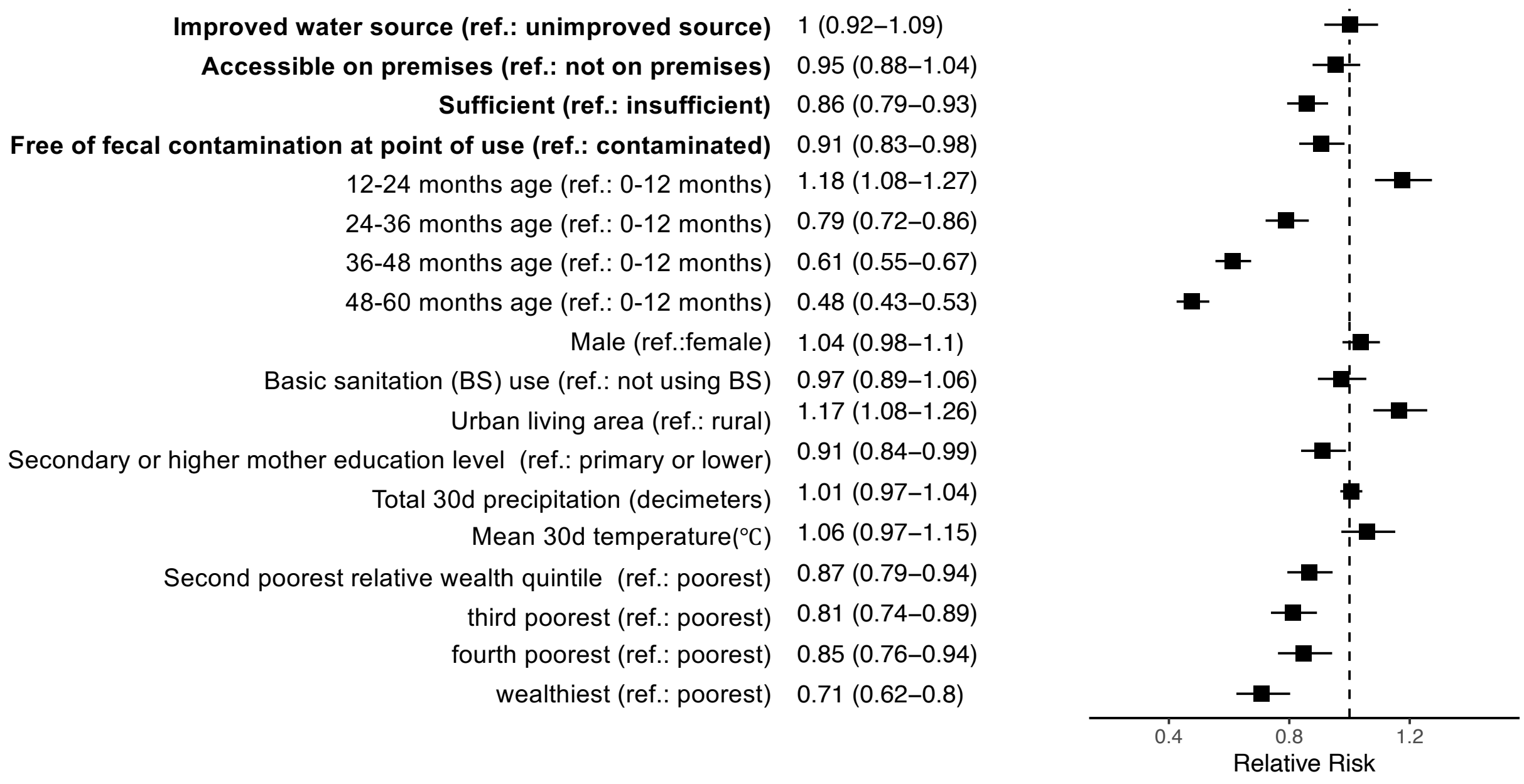

Figure S8**:** Adjusted relative risk of childhood diarrhoea when SMDWS subcomponents are defined using faecal contamination at the POU. Results include adjusted relative risk for diarrhoeal disease in children living in households using a drinking water source which was 1) improved (n=22179, unimproved n=4617), 2) accessible on premises (n= 14992, not on premises n=11804), 3) available when needed (n= 22901, insufficient n=3868) and free of faecal contamination at the POU (n=7989, contaminated n= 18807). Squares represent the point estimates of relative risks and horizonal lines the 95% confidence intervals of the primary exposure (emboldened) and all adjustment variables derived from a multi-level log-binomial generalized linear model. The dashed vertical line at relative risk = 1 marks no change in risk.

	Arid Climate Zone (n=10617)	Temperate Climate Zone (n=4926)	Tropical Climate Zone (n=11557)
**SMDWS use (ref.: not using SMDWS)**	1 (0.89–1.12)	0.86 (0.69–1.07)	0.74 (0.63–0.88)
12-24 months age (ref.: 0-12 months)	1 (0.88–1.13)	1.49 (1.17–1.89)	1.29 (1.15–1.45)
24-36 months age (ref.: 0-12 months)	0.65 (0.56–0.75)	0.92 (0.7–1.19)	0.91 (0.8–1.04)
36-48 months age (ref.: 0-12 months)	0.5 (0.43–0.58)	0.63 (0.47–0.85)	0.73 (0.64–0.84)
48-60 months age (ref.: 0-12 months)	0.41 (0.34–0.49)	0.52 (0.38–0.73)	0.54 (0.46–0.63)
Basic sanitation (BS) use (ref.: not using BS)	0.96 (0.83–1.1)	0.99 (0.79–1.23)	1 (0.88–1.12)
Male (ref.:female)	1.02 (0.93–1.13)	1.13 (0.95–1.34)	1.02 (0.94–1.12)
Urban living area (ref.: rural)	1.11 (0.98–1.25)	1.35 (1.08–1.69)	1.17 (1.05–1.3)
Secondary or higher mother education level (ref.: primary or lower)	0.94 (0.82–1.07)	0.85 (0.69–1.05)	0.91 (0.81–1.03)
Total 30d precipitation (decimeters)	1.05 (0.95–1.17)	1.06 (0.98–1.15)	0.95 (0.9–1)
Mean 30d temperature(℃)	1.08 (0.93–1.24)	0.87 (0.7–1.08)	0.81 (0.68–0.97)
Second poorest relative wealth quintile (ref.: poorest)	0.87 (0.76–0.99)	0.86 (0.66–1.13)	0.9 (0.79–1.02)
third poorest (ref.: poorest)	0.83 (0.72–0.97)	0.75 (0.56–1)	0.81 (0.71–0.93)
fourth poorest (ref.: poorest)	0.83 (0.7–0.98)	0.72 (0.52–0.98)	0.89 (0.77–1.03)
wealthiest (ref.: poorest)	0.71 (0.57–0.88)	0.7 (0.49–1)	0.71 (0.6–0.84)

Figure S9**:** Adjusted relative risk for diarrhoeal disease in children living in households using SMDWS at the POC (n=7320) compared to households not fulfilling all of the SMDWS criteria (n=19476) stratified by children living in arid (n=10617), temperate (n=4926) and tropical climate zones (n=11557). We report point estimates of relative risk and 95% confidence intervals for safe drinking water exposure and adjusted variables.

	Dry Period (n=20162)	Wet Period (n=6634)
**SMDWS use (ref.: not using SMDWS)**	0.89 (0.8–0.98)	0.94 (0.8–1.12)
12-24 months age (ref.: 0-12 months)	1.21 (1.11–1.33)	1.08 (0.91–1.28)
24-36 months age (ref.: 0-12 months)	0.79 (0.71–0.88)	0.78 (0.65–0.94)
36-48 months age (ref.: 0-12 months)	0.64 (0.58–0.72)	0.52 (0.43–0.64)
48-60 months age (ref.: 0-12 months)	0.49 (0.43–0.56)	0.43 (0.33–0.54)
Basic sanitation (BS) use (ref.: not using BS)	0.95 (0.86–1.04)	1.04 (0.86–1.25)
Male (ref.:female)	1.02 (0.96–1.09)	1.09 (0.96–1.23)
Urban living area (ref.: rural)	1.18 (1.08–1.29)	1.1 (0.94–1.29)
Secondary or higher mother education level (ref.: primary or lower)	0.9 (0.82–0.99)	0.95 (0.8–1.13)
Total 30d precipitation (decimeters)	0.99 (0.95–1.04)	1.03 (0.95–1.11)
Mean 30d temperature(℃)	1.05 (0.95–1.16)	1.18 (1.01–1.39)
Second poorest relative wealth quintile (ref.: poorest)	0.92 (0.83–1.01)	0.72 (0.6–0.87)
third poorest (ref.: poorest)	0.81 (0.73–0.9)	0.82 (0.67–0.99)
fourth poorest (ref.: poorest)	0.85 (0.76–0.96)	0.83 (0.67–1.02)
wealthiest (ref.: poorest)	0.7 (0.61–0.81)	0.72 (0.56–0.92)

Figure S10**:** Adjusted relative risk for diarrhoeal disease in children living in households using SMDWS at the point of collection (n=7320) compared to households not fulfilling all of the SMDWS criteria (n=19476) stratified by surveys collected in dry (n=20162) and wet periods (n=6634). We report point estimates of relative risk and 95% confidence intervals for safe drinking water exposure and adjusted variables.

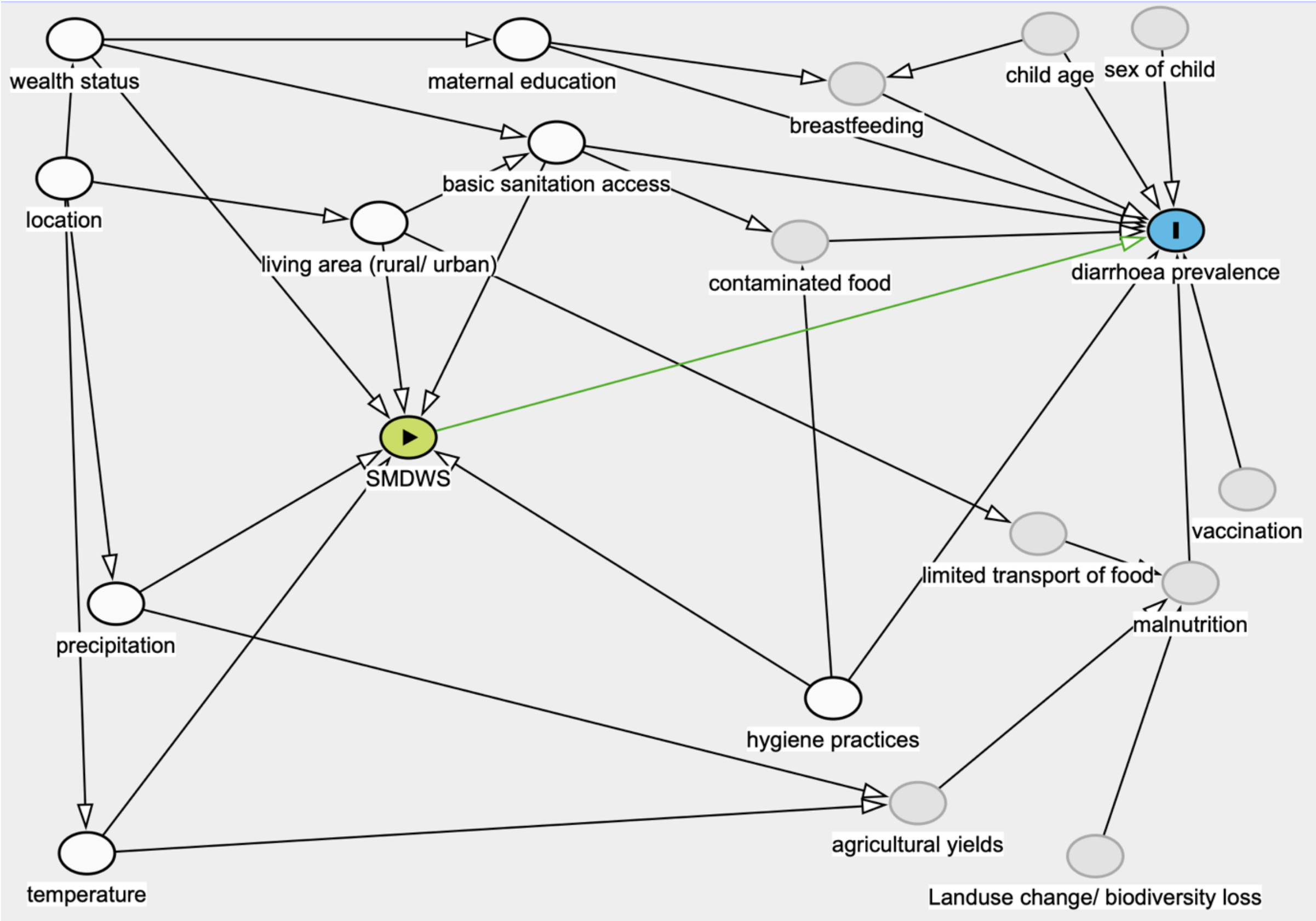

Figure S11**.** Correctly adjusted DAG for estimating the total effect of the SMDWS defined at the POC on diarrhoea prevalence with minimal sufficient adjustment sets containing basic sanitation access, living area (rural/ urban), location, maternal education, precipitation, temperature, wealth status. Accessed on 22 December 2025: https://dagitty.net/m8oHXPBBr

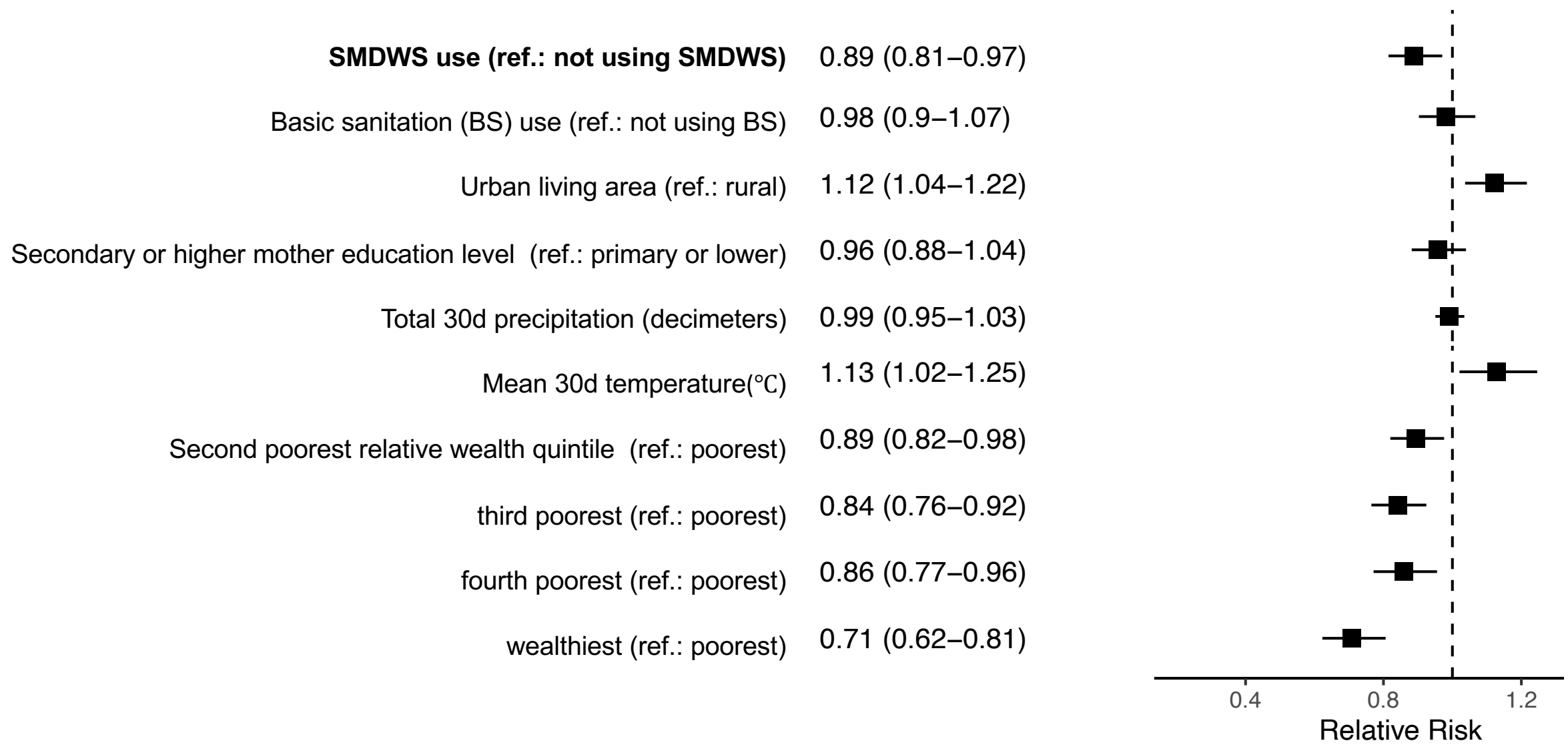

Figure S12**:** Adjusted relative risk for diarrhoeal disease in children living in households using SMDWS at the POC (n=7320) compared to households not fulfilling all of the SMDWS criteria (n=19476) from sensitivity analysis accounting for spatial clustering at subnational level. Squares represent the point estimates of relative risks and horizonal lines the 95% confidence intervals of the primary exposure (emboldened) and DAG informed minimal adjustment variables (Figure S11) derived from a multi-level log-binomial generalized linear model. The dashed vertical line at relative risk = 1 marks no change in risk.

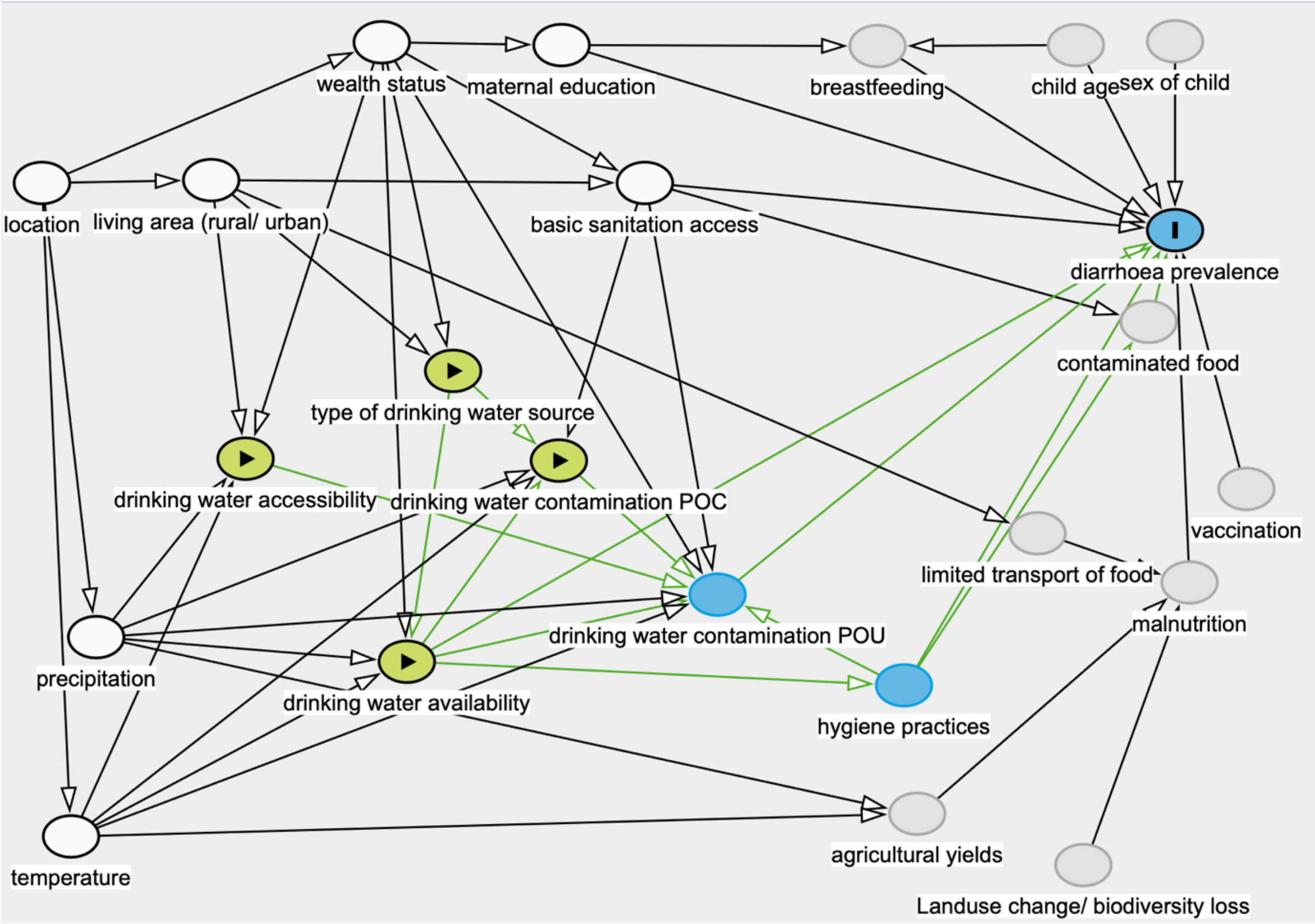

Figure S13. Correctly adjusted DAG for estimating the total effect of the SMDWS subcomponents on diarrhoea prevalence with minimal sufficient adjustment sets containing basic sanitation access, living area (rural/ urban), location, maternal education, precipitation, temperature, wealth status. Accessed on 22 December 2025: https://dagitty.net/m8oHXPBBr

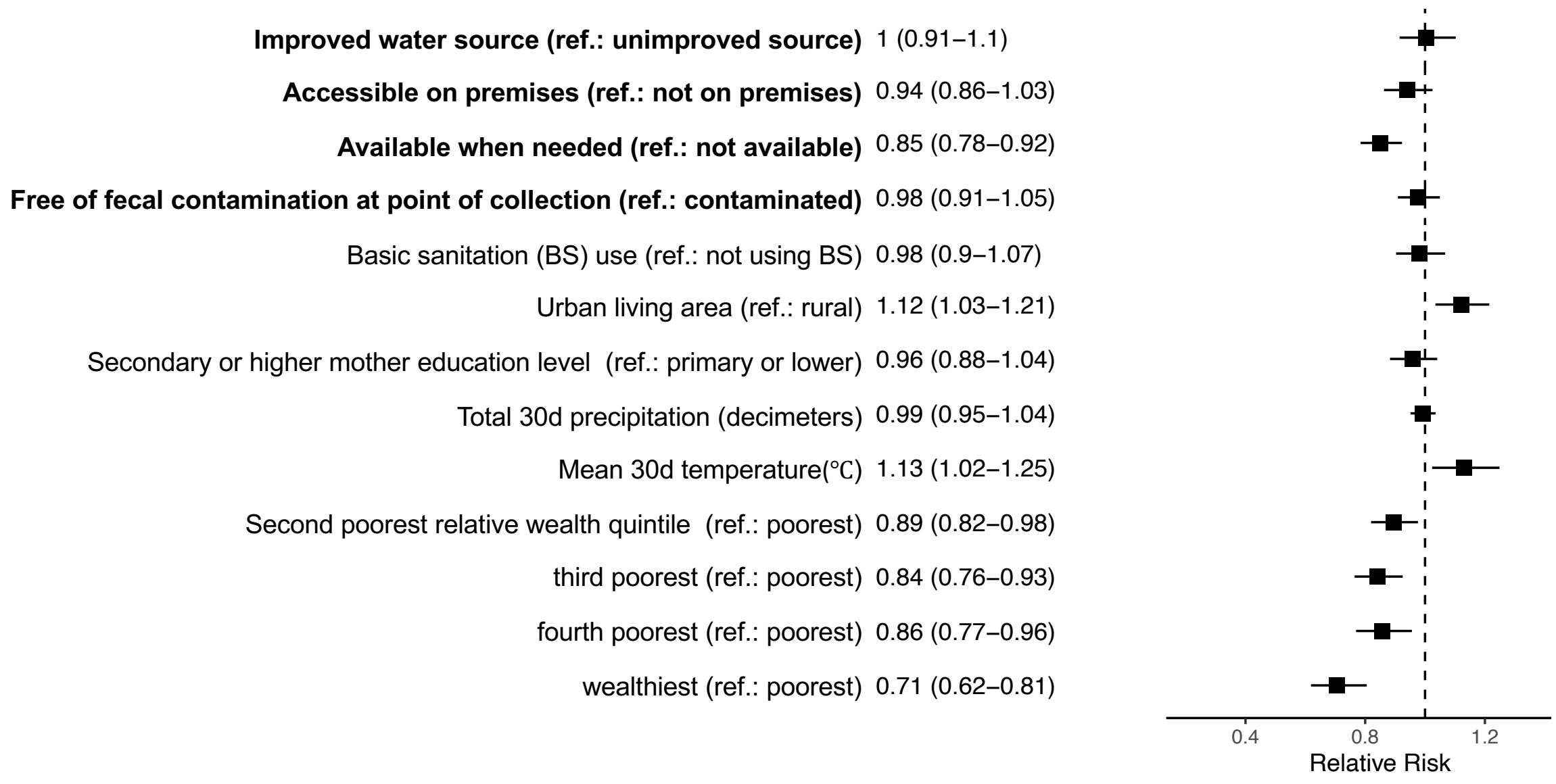

Figure S14: Adjusted relative risk of childhood diarrhoea using a drinking water source which is 1) improved (n= 22179) compared to unimproved (n=4617), 2) accessible on premises (n= 14992) compared to not on premises (n= 11804), 3) available when needed (n= 22901) compared to not available when needed (n= 3895) and 4) free of faecal contamination at the point of collection (n=12582, contaminated n=14187) from sensitivity analysis accounting for spatial clustering at subnational level. Squares represent point estimates of relative risk and horizontal lines the 95% confidence intervals of exposure and DAG informed minimal adjustment variables (Figure S13). The dashed vertical at relative risk = 1 marks no change in risk.

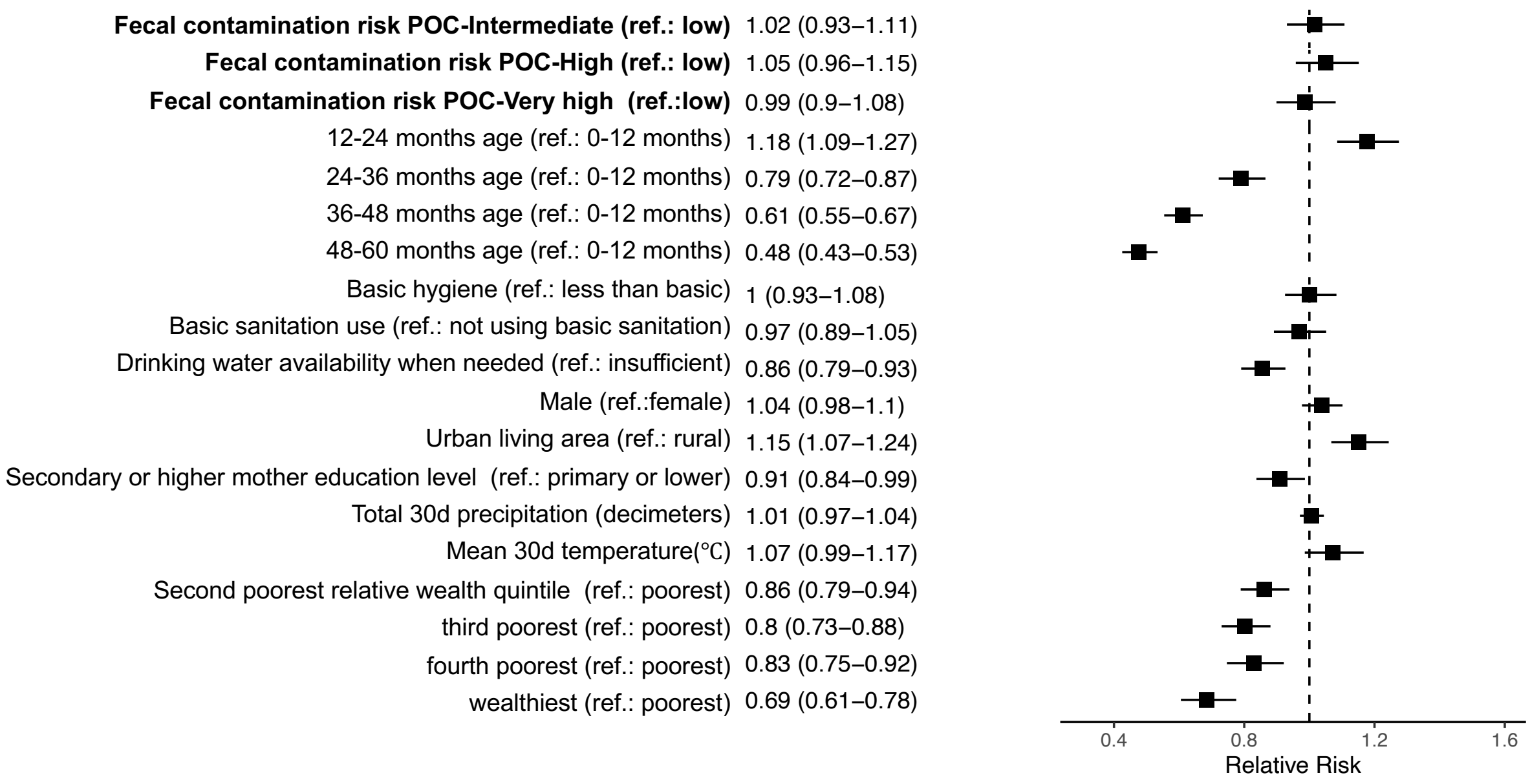

Figure S15**.** Adjusted relative risk of diarrheal disease in children using drinking water contaminated with *E. coli* at different concentrations at the POC (n=26796). Risk categories used reflect the WHO water quality risk categories for *E. coli* with low (<1 colony forming units (CFU)/100ml), intermediate (1-<10 CFU/100ml), high (10-<100 CFU/100ml) and very high (≥100 CFU/100ml) levels of contamination**.** Squares show point estimates of relative risks for each covariate including those of adjusted variables and horizontal lines the 95% confidence interval. The dashed vertical at relative risk = 1 marks no association. Model results are from sensitivity analysis without improved drinking water as an exposure and with basic hygiene. The dashed vertical at relative risk = 1 marks no change in risk.

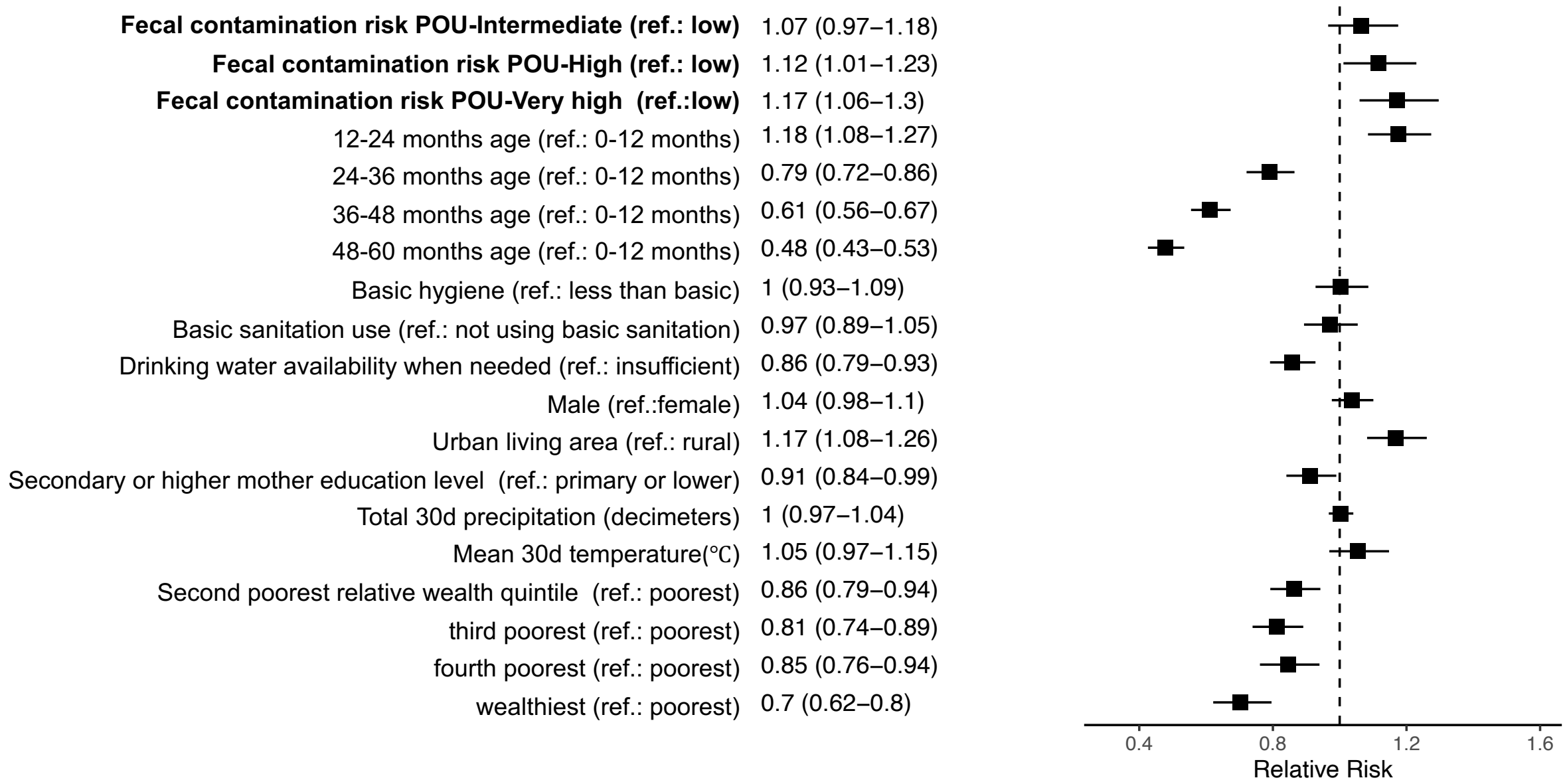

Figure S16**.** Adjusted relative risk of diarrheal disease in children using drinking water contaminated with *E. coli* at different concentrations at the POU (n=26796) from sensitivity analysis without adjusting model for improved drinking water as an exposure. Risk categories used reflect the WHO water quality risk categories for *E. coli*. Relative risks from use of drinking water with intermediate (1-<10 CFU/100ml, at POU n= 5017), high (10-<100 CFU/100ml, at POU n=6058) and very high (≥100 CFU/100ml, at POU n=7732) levels of *E. coli* contamination compared to low levels of *E. coli* contamination (<1 CFU/100ml, at POU n=7989) are plotted**.** Squares represent the point estimates of relative risks and horizontal lines the 95% confidence intervals of the primary exposure derived from multi-level log-binomial generalized linear models. The dashed vertical line at relative risk = 1 marks no change in risk.

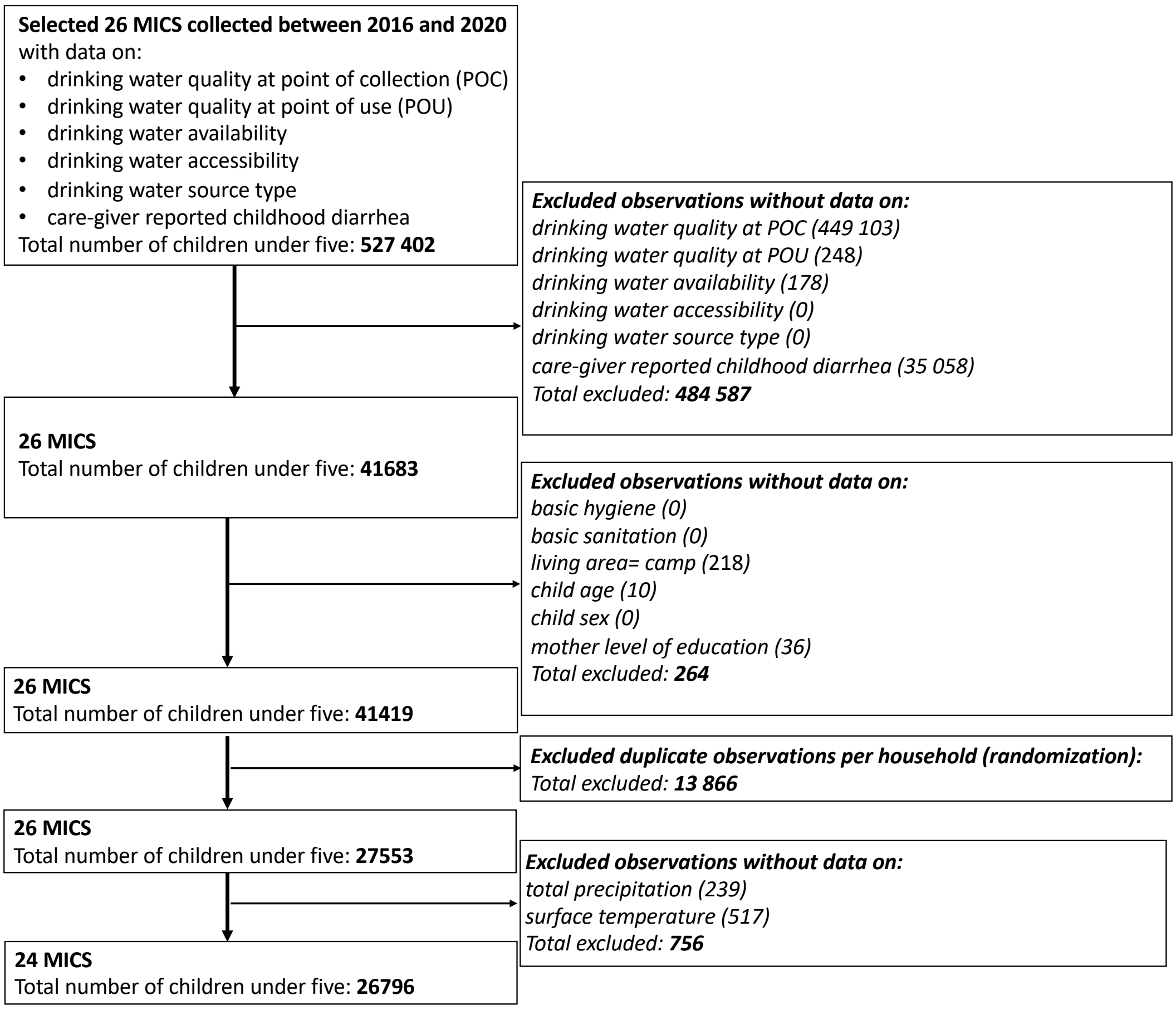

Figure S17: Study flow diagram showing exclusion of observations with missing data on exposure or control variables.

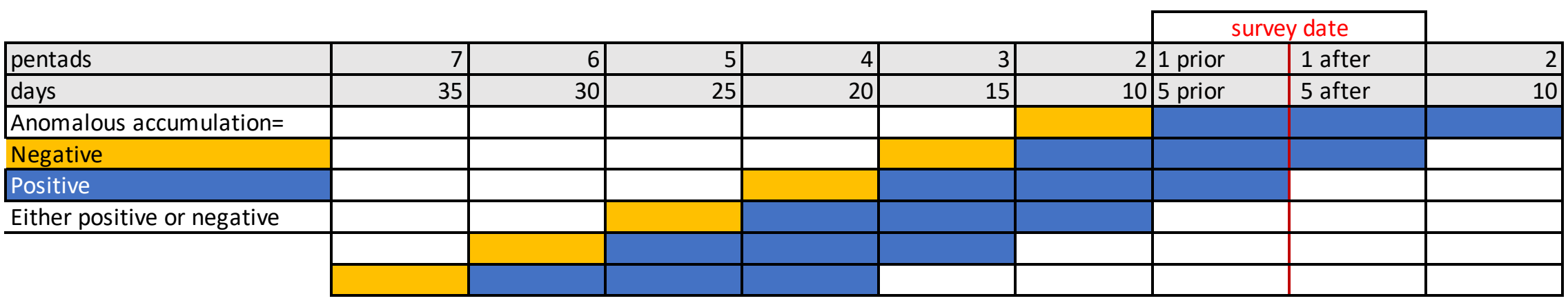

Figure S18: Chart describing all combinations of rainfall conditions accounted for when describing the selection of surveys collected within 30 days of the onset of a wet period which lasted at least 15 days.

Table S1: **Overview of covariates influencing the relationship between safely managed drinking water service use and diarrhoea**

**Variable**	**Analytical role**	**Rational**	**References**
Child age	Precision variable	Diarrhoeal morbidity is influenced by immune development, breastfeeding practices and different exposure pathways at with age.	(Divon & Bergstrøm, 2012; Ssekandi et al., 2023)
Child sex	Precision variable	Biological and social differences may influence susceptibility and care-seeking behaviour	(Mahmud et al., 2020; Ssekandi et al., 2023)
Maternal education	Confounder	Higher level of care-giver education contributes to better household drinking water access and practices and lower diarrheal risk	(Bachrach & Meeks Gardner, n.d.; Edward et al., 2019)
Household wealth	Confounder	Low household wealth status is associated with higher risks for lack of safe drinking water and diarrheal disease	(He et al., 2023)
Living area	Confounder	Rural areas lag behind progress in achieving safe drinking water services. However, rapid urbanization and creation of informal settlements have also been shown to negatively effect water quality management and population health.	(Bain et al., 2014; Dongzagla et al., 2022)
Basic sanitation	Confounder	Lack of sanitation services have been associated with increased risks of diarrhoea in children under five as well as drinking water contamination.	(Joshi & Shrestha, 2015; Overbey et al., 2019; Wolf et al., 2023)

Basic hygiene	Confounder	Handwashing with soap at key times has been shown to reduce diarrhoeal disease. Dirty hands are a risk factor for the microbial recontamination of stored drinking water at the household level.	(Cairncross et al., 2010; Freeman et al., 2014; Pickering et al., 2010)
Average precipitation	Confounder	Precipitation has been associated with both an increase and a decrease in diarrhoea prevalence. Heavy rain that follows dry weather increase diarrhoea prevalence, whereas light showers decrease the diarrhoea prevalence.	(Bhavnani et al., 2014; Kraay et al., 2020);
Average temperature	Confounder	An increase in temperature (or higher mean temperature) has been associated with an increased (or a higher) diarrhoea prevalence.	(Carlton et al., 2016)

Table S2: **Multiple Indicator Cluster Surveys from 24 countries that included data on childhood diarrhoea and safely managed drinking water service variables**.

Country	Fieldwork data collection	Income group	UN region	Climate Zone
**Total**				
Algeria	December 2018–April 2019	Upper middle income	Africa	**Arid**
Bangladesh	January– June 2019	Lower middle income	Asia	**Temperate/ Tropical**
Central African Republic	December 2018– June 2019	Low income	Africa	**Tropical/** Arid
Chad	May– December 2019	Low income	Africa	**Arid/** Tropical
Gambia	January– April 2018	Low income	Africa	**Arid/ Tropical**
Georgia	September– December 2018	Upper middle income	Asia	**Cold**/ **Temperate**
Ghana	October 2017– January 2018	Lower middle income	Africa	**Tropical**
Guinea-Bissau	November 2018– March 2019	Low income	Africa	**Tropical**
Guyana	2019-2020	Upper middle income	Americas	**Tropical**
Iraq	March– May 2018	Upper middle income	Asia	**Arid/** Temperate
Lao PDR	July– November 2017	Lower middle income	Asia	**Temperate/ Tropical**
Lesotho	April– September 2018	Lower middle income	Africa	**Temperate/** Cold
Madagascar	August– November 2018	Low income	Africa	**Tropical/ Temperate**
Mongolia	September– December 2018	Lower middle income	Asia	**Arid/ Cold**
Nigeria	September 2016– January 2017	Lower middle income	Africa	**Tropical**/ Arid
Pakistan Punjab	December 2017– March 2018	Lower middle income	Asia	**Arid**/ Temperate
Paraguay	June– September 2016	Upper middle income	Americas	**Tropical/ Temperate**
São Tome and Principe	August– October 2019	Lower middle income	Africa	**Tropical**
Sierra Leone	May– August 2017	Low income	Africa	**Tropical**
Suriname	March– August 2018	Upper middle income	Americas	**Tropical**
Togo	July– October 2017	Low income	Africa	**Tropical**
Tunisia	March– May 2018	Lower middle income	Africa	**Arid**/ Temperate
West Bank and Gaza	December 2019– January 2020	Lower middle income	Asia	**Temperate/ Arid**
Zimbabwe	January– April 2019	Lower middle income	Africa	**Arid/Temperate**

Table S3: **Results of SMDWS models at POC and POU (RR=Relative risk, CI= Confidence Interval)**

	**At least basic but not SMDWS at POC**	**At least basic but not SMDWS at POU**	**SMDWS at POC**	**SMDWS at POU**
**Less than basic** RR (95% CI)	0.95 (0.88-1.03)	0.95 (0.88-1.02)	0.87 (0.78-0.97)	0.86 (0.76-0.96)
**Not using SMDWS*** RR (95% CI)	-	-	0.90 (0.83-0.98)	0.90 (0.81-0.99)

*SMDWS at POC refers to use safely managed drinking water services which consider absence of fecal contamination at the POC

**SMDWS at POU refers to use safely managed drinking water services which consider absence of fecal contamination at the POU

***at least one of the subcomponent criteria not met

Table S4: **Overview of results from three sensitivity analyses.**

**Exposure**	**Main model**	**Model-S.A.1** RR (95% CI)	**Model-S.A.2** median RR (95% CI)	**Model-S.A.3:** RR (95% CI)
**SMDWS**	0.90 (0.83-0.98)	0.89 (0.81-0.97)	0.91 (0.83-0.99)	0.90 (0.84-0.96)
**Water source type**	1.0 (0.91-1.10)	1.0 (0.91-1.10)	1.02 (0.83-1.12)	1.08 (1.0-1.16)
**Accessibility on premises**	0.95 (0.87-1.03)	0.94 (0.86-1.03)	0.92 (0.85-1.01)	0.84 (0.79-0.89)
**Faecal contamination**	0.98 (0.92-1.06)	0.98 (0.91-1.05)	1.01 (0.94-1.08)	1.0 (0.94-1.06)
**Availability when needed**	0.86 (0.79-0.93)	0.85 (0.78-0.92)	0.85 (0.78-0.92)	0.84 (0.78-0.90)

Main model RR (95% CI): Primary log-binomial mixed model estimating relative risks (RRs) with 95% confidence intervals (CI)

Model-S.A.1: Sensitivity analysis refitting the primary SMDW and subcomponent model (Table S5) using the DAG-informed minimal adjustment set (Fig. S11, S.13) and adding a sub-national district-level random intercept nested within country to account for finer-scale clustering (Results plotted in Fig. S12, S14).

Model-S.A.2: Extension of Model-S.A.1 using Monte Carlo to refit model 100 times on different data sets**,** randomly selecting one child per household; values shown are the median RR and the median of the model-based 95% confidence limits across resamples.

Model-S.A.3: Sensitivity analysis including all eligible children and fitting a marginal (non–random-effects) model with the same covariate set as the primary analysis using robust standard errors clustered at the household level to account for intrahousehold correlation.

Table S5. **Overview of models**

Model	Exposure variables	Adjusted control variables	Outcome variable
**1.1.a (primary)**	• Household use of SMDWS (binary), accounting for faecal contamination at the *POC*	child age, sex of child, basic sanitation access, relative wealth quintile, living area, maternal education level, total precipitation, average temperature, country	Caregiver reported occurrence of diarrhoea in child under five within two weeks prior to survey date
1.1.b	• Household use of SMDWS (categorical), accounting for faecal contamination at the *POC*		
1.2.a	• Household use of SMDWS (binary), accounting for faecal contamination at the *POU*	child age, sex of child, basic sanitation access, *basic hygiene access*, relative wealth quintile, living area, maternal education level, total precipitation, average temperature, country	
1.2.b	• Household use of SMDWS (categorical), accounting for faecal contamination at the POU		
**2.1.a (primary)**	• Faecal contamination at *POC* (binary)<br>• Accessibility on premises (binary)<br>• Sufficiency of drinking water (binary)<br>• Use of an improved source (binary)	child age, sex of child, basic sanitation access, relative wealth quintile, living area, maternal education level, total precipitation, average temperature, country	
2.1.b	• Faecal contamination at *POC* (risk categories)<br>• Accessibility on premises (binary)<br>• Sufficiency of drinking water (binary)<br>• Use of an improved source (binary)		
2.1.c	• Faecal contamination at *POC* (binary)<br>• Accessibility on premises (binary)<br>• Sufficiency of drinking water (binary)<br>• Water source type (categorical)		
2.2.a	• Faecal contamination at *POU* (binary)<br>• Accessibility on premises (binary)<br>• Sufficiency of drinking water (binary)<br>• Use of an improved source (binary)	child age, sex of child, basic sanitation access, basic *hygiene access*, relative wealth quintile, living area, maternal education level, total precipitation, average temperature, country	

2.2.b	• Faecal contamination at *POU* (risk categories)<br>• Accessibility on premises (binary)<br>• Sufficiency of drinking water (binary)<br>• Use of an improved source (binary)		

Table S6: **Tetrachoric correlation coefficients of SMDWS subcomponents**

	Faecal Contamination POC	Faecal Contamination POU	Accessibility on premises	Availability when needed	Improved source
Faecal Contamination POC	1	**0.8**	**-0.33**	-0.054	**-0.7**
Faecal Contamination POU	**0.8**	1	**-0.44**	-0.039	**-0.55**
Accessibility on premises	**-0.33**	**-0.44**	1	0.045	**0.64**
Availability when needed	-0.054	-0.039	0.045	1	-0.015
Improved source	**-0.7**	**-0.55**	**0.64**	-0.015	1

Table S7**:** STROBE Statement—Checklist of items that should be included in reports of ***cross-sectional studies***

	Item No	Recommendation	Page
**Title and abstract**	1	(*a*) Indicate the study's design with a commonly used term in the title or the abstract	1
		(*b*) Provide in the abstract an informative and balanced summary of what was done and what was found	1
**Introduction**			
Background/rationale	2	Explain the scientific background and rationale for the investigation being reported	1-3
Objectives	3	State specific objectives, including any prespecified hypotheses	3
**Methods**			
Study design	4	Present key elements of study design early in the paper	3-4, 11
Setting	5	Describe the setting, locations, and relevant dates, including periods of recruitment, exposure, follow-up, and data collection	3-4, 11
Participants	6	(*a*) Give the eligibility criteria, and the sources and methods of selection of participants	11-12
Variables	7	Clearly define all outcomes, exposures, predictors, potential confounders, and effect modifiers. Give diagnostic criteria, if applicable	3, 11, SI
Data sources/ measurement	8*	For each variable of interest, give sources of data and details of methods of assessment (measurement). Describe comparability of assessment methods if there is more than one group	3, 11
Bias	9	Describe any efforts to address potential sources of bias	3, 7, 11, 12
Study size	10	Explain how the study size was arrived at	11-12
Quantitative variables	11	Explain how quantitative variables were handled in the analyses. If applicable, describe which groupings were chosen and why	11- 15
Statistical methods	12	(*a*) Describe all statistical methods, including those used to control for confounding	3, 11-15
		(*b*) Describe any methods used to examine subgroups and interactions	3, 15
		(*c*) Explain how missing data were addressed	11-12, 15

		(*d*) If applicable, describe analytical methods taking account of sampling strategy	11-12, 15
		(e) Describe any sensitivity analyses	15
**Results**			
Participants	13*	(a) Report numbers of individuals at each stage of study—eg numbers potentially eligible, examined for eligibility, confirmed eligible, included in the study, completing follow-up, and analysed	3
		(b) Give reasons for non-participation at each stage	11-12
		(c) Consider use of a flow diagram	SI
Descriptive data	14*	(a) Give characteristics of study participants (eg demographic, clinical, social) and information on exposures and potential confounders	3-4, Data_S1
		(b) Indicate number of participants with missing data for each variable of interest	SI
Outcome data	15*	Report numbers of outcome events or summary measures	
Main results	16	(*a*) Give unadjusted estimates and, if applicable, confounder-adjusted estimates and their precision (eg, 95% confidence interval). Make clear which confounders were adjusted for and why they were included	4-7, SI
		(*b*) Report category boundaries when continuous variables were categorized	13-14
		(*c*) If relevant, consider translating estimates of relative risk into absolute risk for a meaningful time period	
Other analyses	17	Report other analyses done—eg analyses of subgroups and interactions, and sensitivity analyses	7-8, 15
**Discussion**			
Key results	18	Summarise key results with reference to study objectives	8
Limitations	19	Discuss limitations of the study, taking into account sources of potential bias or imprecision. Discuss both direction and magnitude of any potential bias	10-11
Interpretation	20	Give a cautious overall interpretation of results considering objectives, limitations, multiplicity of analyses, results from similar studies, and other relevant evidence	8-11
Generalisability	21	Discuss the generalisability (external validity) of the study results	10
**Other information**			

Funding	22	Give the source of funding and the role of the funders for the present study and, if applicable, for the original study on which the present article is based	16
